# How Is Equity Considered Within National Healthcare Priority Setting? A Qualitative Study of Australian Health Technology Assessment Actors' Perspectives

**DOI:** 10.1111/hex.70955

**Published:** 2026-10-30

**Authors:** Sheridan Rodda, Melanie Lloyd, Aili Langford, Jedidiah Morton, Zanfina Ademi

**Affiliations:** ^1^ Grad Dip Health Ec and Policy, Health Economics and Policy Evaluation Research group, Faculty of Pharmacy and Pharmaceutical Sciences, Monash University Parkville Australia; ^2^ Health Economics and Policy Evaluation Research group, Faculty of Pharmacy and Pharmaceutical Sciences, Monash University Parkville Australia; ^3^ Murdoch Children's Research Institute Parkville Australia; ^4^ Sydney Pharmacy School, Faculty of Medicine and Health The University of Sydney Sydney Australia; ^5^ Centre for Medicine Use and Safety, Faculty of Pharmacy and Pharmaceutical Sciences Monash University Parkville Australia; ^6^ Baker Heart and Diabetes Institute Melbourne Australia; ^7^ School of Public Health and Preventive Medicine Monash University Melbourne Australia

**Keywords:** decision‐making, equity, health policy, health technology assessment (HTA), inequality, qualitative research

## Abstract

**Introduction:**

Equity is recognised in the decision‐making within Australian health technology assessment (HTA), although limited studies describe how these considerations are operationalised in practice. We aimed to explore the experiences and views of a range of HTA actors, to understand how equity is considered in HTA processes.

**Methods:**

Semi‐structured interviews were conducted with 21 participants including sponsors, members of patient or consumer representative groups, non‐government organisations and interest groups, economists preparing or assessing HTA submissions and people in government‐appointed roles, including HTA committee members. Interview transcripts were analysed using reflexive thematic analysis.

**Results:**

Reflexive thematic analysis produced three overarching themes. Firstly, equity can be considered in decision‐making, but its role is not transparent, and equity meaningfully impacts decision‐making in few circumstances. Secondly, there are institutional, ideological and methodological challenges that impede the consideration of equity in HTA. Ambiguous guidance and public summary documents mean the inclusion, evaluation and appraisal of equity‐relevant information depends on the individual, creating inconsistency. In the absence of a clear equity definition, HTA actors frame equity in ways that serve their own purposes, creating confusion and tension within a reactive process. Decision‐makers face a dilemma between evidence‐based medicine paradigms, which predominate, and patient or lived experience evidence. Limited knowledge of public values on equity priorities constrains decision‐maker ability to integrate equity concerns into recommendations. Thirdly, HTA actors express a preference for incremental and pragmatic HTA reform. They wish to maintain a deliberative approach to decision‐making with qualitative equity information but see scope to adopt strategies such as Citizens' juries or case studies trialling the use of equity‐informed economic evaluations.

**Conclusions:**

This study constructed a detailed understanding of how equity is considered by HTA actors, the challenges faced, and generated potential strategies to better consider equity in Australian HTA.

## 1 Lived Experience or Public Contribution

The focus of the study was to understand the consideration of equity in health technology assessment (HTA), including the role played by consumer or patient contribution. Those with lived experience such as patient and consumer representatives were included as study participants.

Service users, in the context of this research objective, would be policymakers, health‐care providers and economists preparing, evaluating or appraising submissions for HTA. Our research team comprised healthcare professionals, a guideline developer, health economists and a HTA committee member. These authors with service user and lived experience contributed to the study design, analysis and interpretation of data.

## 2 Introduction

Australian health technology assessment (HTA) is centred on the evaluation of the efficacy, quality, safety, effectiveness and cost‐effectiveness of new technologies; however other ‘less readily quantifiable factors,' including equity and ethical principles, are considered within a deliberative process [1, 2, 3, 4]. National healthcare funding decisions have the potential to both ameliorate or exacerbate health inequalities [5]. Equity‐informed economic evaluation (EIEE) methods are emerging that can quantitatively evaluate equity impacts within cost‐effectiveness analysis (CEA), such as distributional cost‐effectiveness analysis (DCEA), or alongside CEA including extended cost‐effectiveness analysis (ECEA) and multi‐criteria decision analysis (MCDA) [6, 7]. Consequently, there is ongoing interest and debate as to how to integrate equity concerns into healthcare priority setting [8].

The well‐established definition for health inequity provided by Whitehead and adopted by the World Health Organization describes differences in health (inequalities) between groups, that are avoidable or amenable to intervention, and are unfair or unjust [9, 10]. Beyond this, inequalities both in health status and in the determinants of health can be considered [11]. Differences can be conceptualised as systematic and associated with disadvantage within social hierarchies [10, 11]. Fairness can mean equal health for all, provided this does not make anyone less healthy, or involve unacceptable trade‐offs against aggregate population health and important non‐health goods [12]. Furthermore, health equity can incorporate fairness of (HTA) procedures, which has been operationalised using the A4R framework [13, 14, 15]. As widely used in health economics [16], in this study we define health equity as the absence of differences in health outcomes seen across population groups that are avoidable and unfair. Health inequities are determined by the conditions in which people are born, grow, live, work, play and age, as well as biological determinants. Populations experiencing disparities can be operationalised using the PROGRESS PLUS acronym (Place of residence, Race/ethnicity, Occupation, Gender, Religion, Education, Socioeconomic status, Social capital, “Plus” additional elements such as age, sexual orientation, disability or other vulnerabilities [17, 18, 19].

Australia's HTA process is complex and described in detail in Supporting File 1 and Figure 1. Two independent, expert HTA bodies are appointed responsibility for evaluating new technologies for Federal government subsidy. The Pharmaceutical Benefits Advisory Committee (PBAC) assesses medicines and vaccines for listing on the Pharmaceutical Benefits Scheme (PBS), and the Medicare Services Advisory Committee (MSAC) evaluates medical technologies and services provided through the Medicare Benefits Schedule (MBS). Sponsors submit applications to the Department of Health (DoH) for funding, which undergo an interactive process between government‐contracted evaluators, sponsors, and HTA subcommittees. Additionally, the Australian Technical Advisory Group on Immunisation (ATAGI) advises PBAC on vaccines [20]. PBAC and MSAC decisions are provided to the Minister for Health, Disability and Ageing, followed by financial agreement negotiations between the sponsor and government. Public summary documents (PSD's) are regularly released to transparently communicate the recommendations made [21].

**Figure 1:**
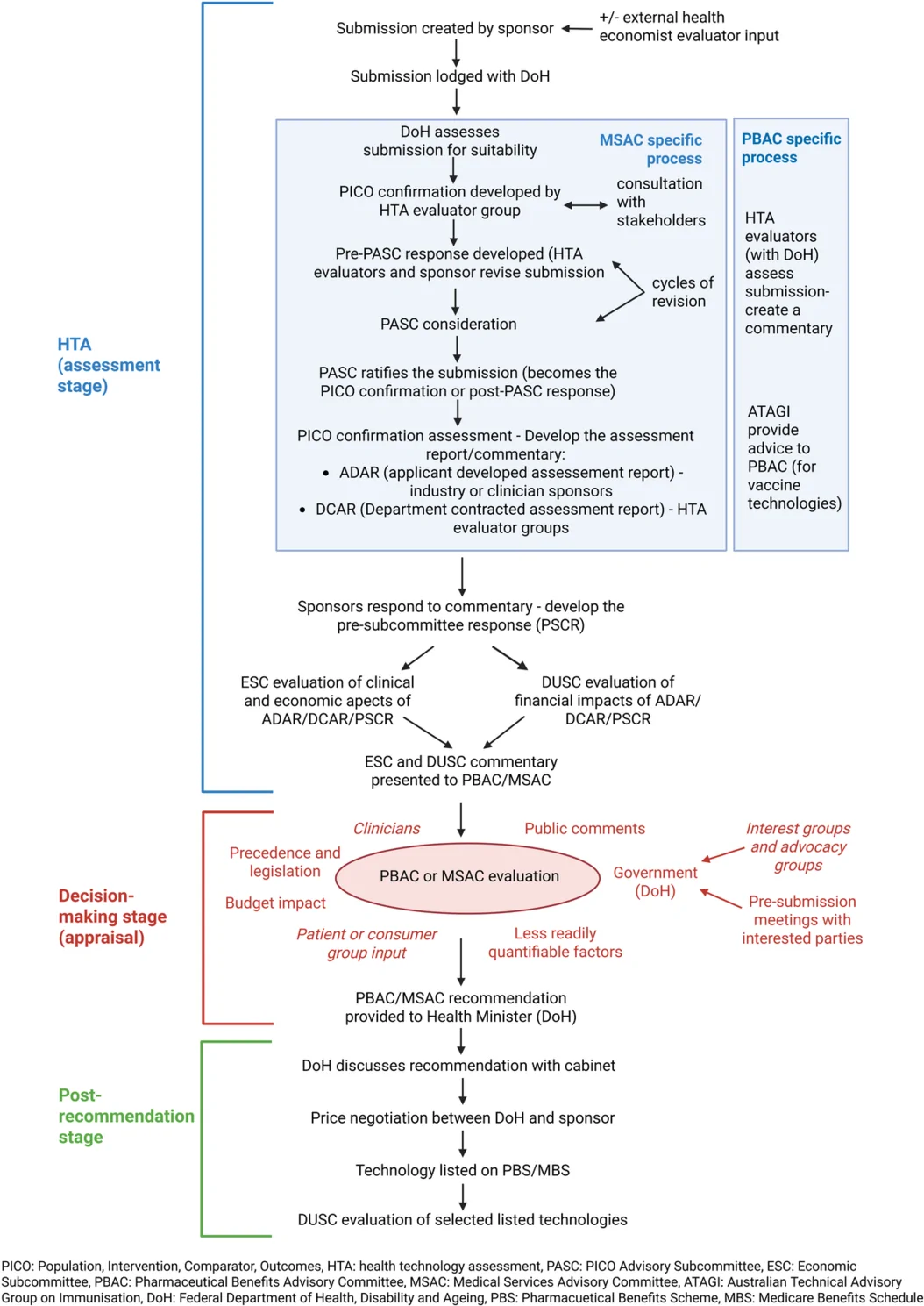
The HTA process.

The integration of equity into HTA processes has proven elusive to demonstrate and difficult to implement in practice. The 2022 Australian Government‐commissioned HTA review found that broader value elements, such as equity, are considered informally and qualitatively, through a deliberative process, with limited transparency in how this evidence informs decisions [22, 23]. Analysis of PSD's identified strong revealed preferences for equity among decision makers, particularly for the health gains of children [24]. Equity is mentioned in PSD's for vaccines and where equity‐specific subgroup analysis has informed decisions [21]. However, the role that equity factors play within these decisions cannot be clearly determined from PSD's and published policy documents do not entirely reflect decision‐maker and government actions [24, 25]. Information is limited as to how HTA bodies operationalise these ‘less‐readily quantifiable factors.' [2, 21, 24] Understanding the cultural and institutional context of decision‐making can support change towards more equity‐informed decisions [26].

Sellars et al. explored how current and former PBAC or MSAC members consider these less quantifiable factors in decision‐making [2]. They confirmed equity is considered implicitly for Aboriginal and Torres Strait Islander populations, out‐of‐pocket expenses and geographical differences in access to treatment, and that further research is required into how equity concerns influence decisions [2]. Li et al. compared this same dataset to earlier decision‐maker interviews (2014), to explore how decision‐making related to the A4R framework over time [27]. Equity concerns were perceived to be of importance, considered qualitatively in a context‐specific manner and included unmet clinical need, illness severity, equity of access, and other aspects of disadvantage [27]. However, the A4R framework, is centred on procedural fairness, with limited and implicit direction on distributional justice [27]. Australian HTA bodies have incorporated the A4R criteria, prioritising procedural fairness, while knowledge gaps remain in their handling of distributive equity [27]. Further they observed equity and ethical implications may only surface late in the decision‐making stage, while it is preferable to identify these issues prior to PBAC or MSAC [27]. Therefore, understanding the pre‐appraisal stage is critical. Both studies used interviews focussed on HTA for children and young people and only included HTA decision‐makers which limits the transferability of these findings [2, 27].

To integrate EIEEs into HTA, we still need to understand: which methods are relevant for HTA actors, for whom they have equity concerns and whether equity analysis is useful on a ‘case‐by‐case basis,' given feasibility constraints [7, 23, 26]. This research must include all relevant stakeholders (actors from both HTA and decision‐making stages) to create more contextualised findings [7, 28]. We are missing a comprehensive picture of how equity of all types (procedural, distributional or otherwise) is operationalised across all HTA stages from a diverse range of perspectives. These questions are timely as we await the government response to the HTA review and advice from HTA Review Implementation Advisory Group [29]. We aimed to explore the experiences and views of HTA actors to better understand how equity is incorporated and considered in Australian HTA and how these approaches may be strengthened in the future.

## 3 Methodology

### 3.1 Study Design

This qualitative study comprised semi‐structured interviews with a diverse range of HTA actors. We adopted a critical realist perspective with a contextualist epistemology to acknowledge that reality can only be partially accessed, as it is shaped by individuals' experiences within their social context [30, 31]. We reported this study according to the Reflexive Thematic Analysis Reporting Guidelines (RTARG) [32].

### 3.2 Participants and Recruitment

Eligible participants were actors currently involved in HTA policy formulation or decision‐making (e.g. PBAC and MSAC committees) at the National level. This included people in government appointed roles, such as members of HTA committees and subcommittees, people representing or working within the pharmaceutical or health technologies industry, members of patient or consumer representative groups, HTA evaluators, members of health‐related non‐government organisations and academic experts [26, 33].

Purposive sampling with snowballing was used to ensure adequate participant diversity. Participants were identified via publicly available information and recruited via email. We applied the concept of information power throughout data collection to evaluate sample size across five dimensions: study aim, sample specificity, application of theory, quality of dialogue and analysis strategy (see Supporting File S2) [34].

### 3.3 Data Generation

Existing qualitative literature in healthcare priority setting and our research questions guided development of the semi‐structured interview guide (Supporting File S3). This covered: experiences of incorporating equity in HTA, definitions, motivations and opinions regarding equity in HTA and how equity might be further integrated into HTA. We pilot tested the guide with a health economist familiar with the Australian HTA context. SR conducted interviews via video call during May to October 2025. Interviews were transcribed and checked verbatim by SR and de‐identified prior to analysis, see Supporting File S2 for details.

### 3.4 Analysis

Transcripts were analysed with NVivo 15 (QSR International) using reflexive thematic analysis [35, 36] with an experiential orientation. In brief, SR and ML familiarised themselves with the dataset then SR conducted predominantly inductive coding. Codes were reviewed and refined before a second round of coding. SR constructed initial themes from the finalised codes. Themes were reviewed by ML to ensure they adequately reflected the dataset then refined by the research team (SR, ML, AL, JM, ZA) to ensure the themes were coherent. Cross‐case analysis by participant types was considered but not undertaken, because meaningful and systematic patterns in experiences and perceptions were not observed in the dataset and to maintain confidentiality for study participants. (see Supporting File S2 for details).

### 3.5 Reflexivity

Our research team comprised a diversity of genders, career experience and backgrounds (health practitioners, guideline developers, health economists). We acknowledge our existing interest in the integration of health equity into HTA. Nonetheless, we approached study design and analysis with genuine curiosity and a desire to generate new knowledge irrespective of whether this supported our research agenda. SR used a reflexive journal during data collection and analysis to maintain critical awareness interactions with participants and the data. See Supporting File S2 for a detailed reflexivity statement.

### 3.6 Ethical Considerations

This study was approved by the Monash University Human Research Ethics Committee (ID 46607). All participants were anonymised for data analysis and reporting.

## 4 Results

We invited 36 individuals to participate: 7 declined, 8 did not respond and 21 proceeded to complete interviews. Participants spanned a variety of sectors as described in Table 1. Interview transcripts had a mean duration of 37 min (range 20‐57 min). The final sample size was found to hold sufficient information to meet the research aims and was in line with similar studies [2, 37, 38]. We developed three overarching themes described below and illustrated in Figure 2. A detailed description of the themes with supporting quotes are provided in Supporting File S4.

### 4.1 Equity Impact on Decision‐Making is Limited and not Transparent

Decision‐makers described possessing flexibility for equity to influence decisions within legislative constraints. Participants felt equity issues are regularly raised in deliberations, but the impact of equity was not meaningful in many instances.

It's more like we have to acknowledge that this is an equity issue, but that's as far as it goes. Is my experience.” “there'll be a discussion that there will be some access barriers for people who live further away from those kind of centres. Is there anything we can do about that? No….

Participant_8

Equity concerns for Aboriginal and Torres Strait Islander peoples, rural or remote populations, children and rare diseases were perceived to have dominated discussions, but actors also expressed concerns for women.

Participants described limited visibility of the role equity played in decision‐making, which was attributed to the deliberative, multifactorial nature of the decision‐making process. Many inputs to the deliberative process (including equity) were described (Figure 1), and these were perceived as open to external influence via relationships and stakeholder engagement processes. External influences were thought to impact both HTA and federal funding allocation for health programmes, where the ‘loudest voices' (disease‐specific, well‐organised and well‐resourced advocacy groups) receive more attention and funding. These groups can use arguments for equity of access to treatments to support their advocacy, though in this case their definition of equity may not be consistent with that which is considered socially determined, avoidable or unfair (Theme 2.6). Conversely, participants were concerned that underrepresented or disadvantaged populations have less capacity to advocate for themselves which was seen to be inequitable.

…the louder voices, the people who are more articulate and are better organised and are in that sort of higher SES group who have greater kind of, societal kind of connections are able to…influence things a lot more.

Participant 17

**Table 1:** Study participants.

Category	Description	Number of participants (*n* = 21)
Bureaucracy	People in government‐appointed roles including HTA committees (PBAC, MSAC, Medical Devices and Human Tissue Advisory Committee) or their subcommittees	7
Industry	People representing or working within the pharmaceutical or health technology industry Health economists working with industry to construct submissions for HTA	5
Patient or consumer representatives and NGO's	Senior members of: Patient or consumer representative groups Health‐relevant non‐governmental organisations	4
HTA evaluators	Health economists contracted by the government to construct or assess submissions for HTA	3
Academics	Academics conducting research relevant to the integration of equity in HTA	2

*Note:* Some participants could be described by > 1 category but were classified by the role that was central to their discussion in interviews.

**Figure 2:**
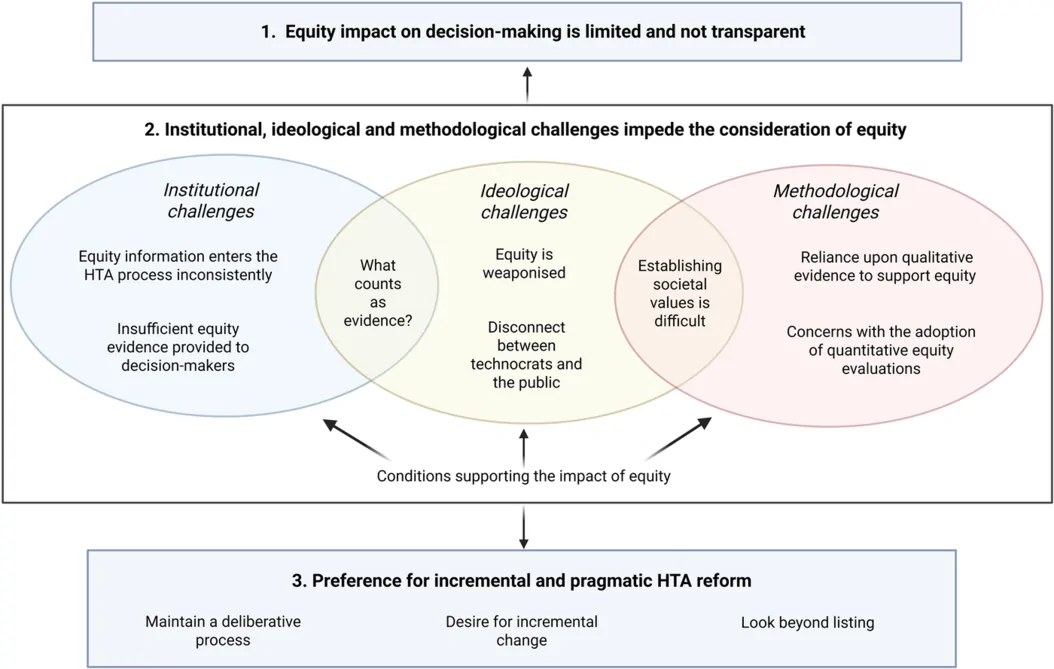
Overview of themes.

### 4.2 Institutional, Ideological and Methodological Challenges Impede the Consideration of Equity

#### 4.2.1 Equity Information Enters the HTA Process Inconsistently

Equity information was described as entering the HTA process organically and in a context‐specific manner via multiple routes. Participants expressed contrasting opinions on whose role it is to incorporate equity concerns, encompassing all HTA actors. Furthermore, participants reported that unclear HTA guidance on how equity information should be submitted, assessed or appraised leaves its incorporation open to interpretation. How equity is integrated was seen by participants to depend on individual beliefs, awareness, work context, and lived experience.

It just creeps in and it's very dependent…. …The things that get brought in like equity, I think would be very dependent on who looked at it and possibly even who within that evaluation team.

Participant_5

Participants preparing submissions expressed a desire to raise equity concerns, but previous experiences, where equity evidence had been disregarded, has resulted in apathy and scepticism that decision‐makers would genuinely consider quantitative equity analyses.

HTA evaluators held conflicting views on the incorporation of equity. It was seen both as the evaluators role to bring attention to equity concerns due to their training, background, experience or team, and alternatively, that their role was to focus on safety, effectiveness and cost‐effectiveness. Further, participants conveyed a distinction between decision‐makers who possessed agency to actively incorporate equity, and actors in the preceding HTA process who feel the HTA guidelines do not empower them to add equity information. All evaluators were however willing to include equity analyses if this was requested and they were resourced to do so.

Actors that were not decision‐makers, perceived that individual biases or ideological views of decision‐makers could influence or shape their appraisal, and that decision‐makers may not be representative of the public. Participants noted the predominance of clinicians in HTA committees whose perspectives may impact equity consideration (focus on access to new and effective treatments, limited understanding of economics and equity‐informed approaches).

it's left to the Department of Health and a select member of individuals on a committee. Which may or may not be representative of the Australian population.

Participant_19

Lastly, participants described that PSDs provide insufficient detail about how equity information was applied to the decision. Participants explained that with this absence of feedback, sponsors may be disinclined to produce equity information, and HTA process actors cannot improve their provision or assessment of equity information.

#### 4.2.2 Insufficient Equity Evidence Provided to Decision‐Makers

The equity‐relevant information provided is perceived as insufficient for decision‐makers to consistently and extensively consider equity impacts. For instance, patient experience evidence was viewed as essential to considering equity, but inadequate, with concerns this evidence was not always representative or reflecting those truly affected, potentially exacerbating inequities.

Participants described producing high‐quality and representative patient experience evidence as challenging and resource intensive. Patient organisations face difficulties engaging with the HTA process due to short timeframes and limited transparency of upcoming agendas.

you know the time challenge would be avoided if patient organisations were involved much earlier in the process.… unless someone tells us, we're not aware until we actually see the agenda of what's being considered.

Participant_10

#### 4.2.3 What Counts as Evidence?

Decisions around what constitutes suitable evidence and what data we collect, were perceived to shape the way equity can be considered. Participants felt bound to follow the HTA guidelines, excluding evidence as prescribed, while some described feeling constrained by the guidelines. Participants described conflict regarding what should constitute evidence within HTA. Many actors perceived that decision‐makers and government hold a restrictive view about what types of evidence receive serious consideration, with precedence given to high‐quality, quantitative data.

because evidence‐based medicine is all randomised studies and it's hard for us to present anything to clinicians that is not randomised, because they don't, they can't conceive non‐randomised evidence as being worthy or of at the same level.

Participant 4

Real‐world evidence, patient narratives or qualitative studies were seen to be given less weight in decision‐making. Participants perceived these evidence types were deprioritised, particularly by clinicians who follow a biomedical paradigm of health with RCTs as gold standard. Furthermore, one participant thought decision‐makers may choose what constitutes acceptable evidence based on what they can evaluate and understand.

if somebody presents something new, somebody's got to be able to evaluate it. [laughs] And so if nobody on the committee goes, has any experience with it, then it's likely to be discounted. Yeah, because you can't interpret it.

Participant_15

Decisions around what counts as evidence were also perceived to also influence the types of treatments and evidence pursued in the future. For example, the focus on RCT data for curative treatments, especially cancer, may leave some therapeutic avenues underexplored and the de‐prioritisation of qualitative evidence may result in less research examining patient preferences and experiences. Lastly, participants noted that what's measured matters and there is insufficient equity data to support equity evaluations. Choices made by the government as data custodians, were perceived to impact the way inequalities can be considered.

#### 4.2.4 Equity Is Weaponised

Participants described how the reactive, adversarial HTA system may generate inequity. It responds to sponsor submissions rather than proactively pursuing health equity goals, its complexity is a potential disincentive for sponsors thereby exacerbating perceived inequity of access issues, and its reliance upon sponsors for information limits our ability to assess equity impacts. Participants perceived that tensions may exist between the government and sponsors as industry utilises existing rules to tailor HTA applications to their advantage (termed ‘gaming the system').

Equity concerns were held for a large variety of social groups with no consensus as to which should be prioritised. These groups were perceived to compete for priority and attention amongst policymakers. Participants believed HTA guidance does not provide a clear definition of equity, creating an opportunity for actors or groups to frame ‘equity' in ways that serves their own purposes.

Four different frames or definitions of equity in HTA were described. 1) ‘Equity of access to innovative treatments' to compensate for unfair future quality‐adjusted life year (QALY) losses due to disease severity, rarity, childhood or high unmet clinical need. This was a dominant frame used commonly by sponsors, clinicians and patient or consumer representatives. 2) ‘Equity of access to care' is considering treatment pathways and potential barriers to accessing funded technologies. For instance, decision‐makers may consider geographical, ethnic and socioeconomic barriers. 3) ‘Social equity' or aiming to compensate for baseline disparities in health across social groups was the least dominant frame. 4) ‘Equity of decision‐making' was applied by decision‐makers aiming to make fair, consistent decisions.

because of the nature of…the HTA system that we're in, where it's quite combative, I guess equity gets weaponized in those regards, like weaponized from a sponsor perspective to try and say, you know, fund this particular thing. And then because, you know, it's an equity issue [nervous laugh] if you don't fund it.

Participant_13

Within the reactive, adversarial HTA context, the use of these opposing equity definitions can create tension and frustrations that make equity discussions challenging. Participants explained how actors may construct equity narratives to support listing a technology, e.g. equity of access for rare diseases applied to specialised treatments for small diagnostic subgroups. The ‘weaponisation' of equity was described where social norms on the importance of equity may be used persuade decision‐makers. Conversely, participants recognised that the DoH and decision‐makers have obligations for transparency, fairness and consistency, meaning these equity narratives may be perceived as unclear, irrelevant or inconsistent with HTA guidance. The adoption of EIEEs was perceived as risky due to this potential for strategic framing.

I think there's a real risk, whenever you have explicit rules and companies, there'll be gaming against those explicit rules, and so…I think the gaming side of things is always something you've got to think about in this market‐based, you know, sponsor‐driven approach. So that's probably my main concern about having more explicit quantitative processes.

Participant 21

#### 4.2.5 Disconnect between Technocrats and the Public

HTA decision‐makers were seen as technocrats appointed by the government to act on behalf of the public interest. Participants believed that to reflect societal values, the public's voice could reach technocrats through estimation of societal preferences or via government and elected representatives. The Government was seen as supporting health equity, but its directives were perceived by many HTA actors as too weak to confidently integrate equity into decision‐making. A variety of participants perceived this lack of established equity priorities and unclear government directives as an information disconnect between technocrats and the public.

I think we've got a disconnect between society and their voice through politicians, the technocrats, if you like, that sit within this kind of space, and then the research to actually be able to provide the support to the technocrats that actually links back to the public preferences or their voice through parliamentarian process.

Participant 3

Consequently, the value HTA actors place on equity and outcomes may not align with the public values. Facing this imperfect information, a decision‐maker described discomfort in making assumptions on how to value equity. This fear of overreach was perceived to weaken the influence of equity concerns on decisions.

that's the biggest barrier really… it stops you, you know, I'm very concerned about um… stereotyping or placing… my opinion over, you know, what might be. And so I think it does sometimes, you hesitate sometimes to go too heavy on the equity aspect because of that.

Participant_5

#### 4.2.6 Establishing Societal Values Is Difficult

Determining societal preferences was depicted by participants as methodologically and politically challenging. They described mathematical methods such as discrete choice experiments, which aggregate preferences over a sample population. However, participants held reservations about their construction, robustness, transparency, representativeness and static nature. Citizen juries were seen to have a potential role in understanding public preferences via a deliberative process. Participants felt equity trade‐offs in HTA should be understandable to the public, but poor public understanding of HTA means considerable effort would be required to support public engagement.

Participants noted the public may hold wide‐ranging beliefs about equity and the use of taxpayer dollars, making agreement on equity priorities politically challenging. Furthermore, as equity was seen to depend on the current social and political environment, participants expressed unease that social norms or stigma around which equity concerns are ‘worthy' may perpetuate inequalities for underrepresented communities. Lastly, participants expressed caution in determining public preferences, as once established, we are obliged to follow them, even if they don't align with our own perceived equity values.

But whatever process it has, it has to be genuine. And so if the population says something vastly different, or different to those who have well experienced in this area, are we prepared to shift values in response to the population? Because I think it's quite challenging.

Participant_7

#### 4.2.7 Reliance Upon Qualitative Evidence to Support Equity

Currently equity evidence is predominantly provided qualitatively. Both decision‐makers and other HTA actors acknowledged qualitative equity evidence may be less explicit or informative, therefore having have less impact on decisions compared with quantitative evidence.

I think qualitative can be done, you know, you could see comment on things, but I think often in that qualitative sense, it often doesn't lead to action.

Participant_16

Participants from industry or patient/consumer organisations felt that patient experience evidence needs greater recognition in decision‐making to better evaluate equity impacts. Where qualitative evidence is available, it was seen as poorly integrated into evaluations due to a lack of capacity, skills or directives. Participants perceived that decision‐makers therefore rely upon commentary from representative groups at deliberation as a proxy for more informative evidence within the assessment.

#### 4.2.8 Concerns With the Adoption of Quantitative Equity Evaluations

Participants conveyed a reluctance to adopt quantitative equity evaluations. Many lacked confidence in the equity weights underpinning DCEA and weighted MCDA due to their derivation, limited understanding among decision‐makers, inability to account for intersectionality, the potential for preferences to change over time, and the need to set an ICER threshold.

The male‐women, I think, is a classic…If we ask the questions in the 1930s, 50 s, versus asking now, they're very different. So changing societal preferences, …we don't want to necessarily lock ourselves into kind of social preferences of a particular cohort and then carry that forward.

Participant_3

Participants expressed concerns that quantitative equity evaluations, especially DCEA, would further complicate an already complex HTA system, exacerbating issues with timely access to technologies. Participants described that when facing increased complexity, decision‐makers may misinterpret or give less attention to equity evaluations. Potential unintended consequences were of concern to many participants whereby quantitatively prioritising some groups could inadvertently disadvantage others, potentially worsening inequities, especially for underrepresented populations.

Data limitations were seen as a key barrier, particularly for females, paediatric populations and Aboriginal and Torres Strait Islander peoples. Participants thought these data gaps would increase uncertainty and therefore reduce the perceived robustness and impact of EIEEs. DCEA particularly was seen as complex, less transparent or less influential than qualitative approaches. Participants viewed EIEEs as less feasible than qualitative assessments due to the added time, costs and resources needed, limited HTA workforce capacity and the training required to adopt methods such as DCEA.

#### 4.2.9 Conditions Supporting the Impact of Equity

Participants agreed equity meaningfully impacts decisions in some instances, citing vaccine listings for Aboriginal and Torres Strait Islander people. Participants described specific conditions where equity was impactful. First, the focus is on a clearly defined social group where equity concerns are widely agreed‐upon. Second, quantitative evidence including differential uptake, effectiveness and disease burden across social groups was included. Third, as ATAGI uses the ‘GRADE evidence to decision framework,' the structure supporting appraisal and evaluation for vaccines explicitly includes equity as a criterion. Fourth, vaccine funding structures allow for targeted equity‐focused decisions through differential listings for specific subgroups.

I mean, there's lots of examples of vaccines, obviously, a lot of the programmes are designed with equity considerations in mind, and in particular, as an example, ah, funding for ah eligibility criteria for vaccines for Indigenous people is often a small but important subgroup.

Participant_1

Finally, participants acknowledged it is easier to value reducing inequalities when budget impact is modest, as for relatively small Aboriginal and Torres Strait Islander populations.

### 4.3 Preference for Incremental and Pragmatic HTA Reform

#### 4.3.1 Maintain a Deliberative Process

Participants wished to maintain a deliberative process, preserving flexibility for decision‐makers to consider equity. Continuing to use qualitative equity evidence was perceived as more palatable, potentially avoiding the issues with EIEEs and political risks with equity weighting.

I think that [pause] there is a political sensitivity associated with a more mathematical way of equity weighting. So if we actually say, we value QALYs that accrue to a particular population 50% more than we do to another group, and that gets rubber‐stamped by government, that is a political risk… handling it more qualitatively, avoids that specific risk…

Participant_12

Conversely, some participants suggested, or were receptive to, a range of approaches to quantitatively evaluate equity (Table 2). For instance, ECEA (e.g. evaluating the out‐of‐pocket costs of healthcare alongside cost‐effectiveness analysis), had been applied by participants in other health policy contexts and was seen as more flexible than DCEA by avoiding normative judgements.

#### 4.3.2 Desire for Incremental Change

While the HTA review was viewed as a catalyst for improving equity consideration, participants acknowledged that maintaining qualitative inputs within a more structured deliberative process represents incremental change. Participants described various reasons for this pragmatic approach: urgency (taking imperfect ‘baby steps' as significant HTA reform will take time), potential for political ramifications or unintended effects on industry, decision‐makers feeling uncertain or challenged in how to respond to more informative equity evidence, and risk‐averse actors constrained by legislation, precedence and public or stakeholder perceptions.

Participants provided conflicting opinions between the mandatory consideration of equity information in every HTA submission (for systematic and fair processes), and concerns that this additional burden may deter sponsors from entering the Australian market. Ways to potentially address this conflict were offered (Table 2).

PBAC and MSAC decision‐making (pauses) is influenced by precedence, …and then trying to keep consistency in decision‐making, because that's where you sort of…that's when you get into trouble. You can't say, well, we…formally considered equity as an issue here, but we're not gonna consider it here. Once you've done it, then you have to be prepared to accept that information and demonstrate that you've looked at it in a consistent way as you go forward.

Participant 15

Alternatively, some participants argued for wider HTA reform including evaluation of a wider range of technologies and adopting a proactive approach where an equity‐focussed agenda directs healthcare funding.

#### 4.3.3 Look Beyond Listing

While discussions focused on PBAC or MSAC, participants agreed that we must also look beyond the decision to list technologies for funding to reduce health inequalities. Participants described how an equity lens should be applied to the entire process from agenda setting, in pre‐HTA safety and efficacy data, through the administration of the PBS (restrictions, retention of technologies or delisting and disinvestment decisions) to the evaluation of real‐world usage post‐listing (Table 2).

Federal (and state) health programme funding decisions (outside PBAC and MSAC) were also considered important to equity. This HTA was seen as less structured, with equity more freely integrated, but also less transparent with limited HTA capability. Participants perceived these processes to have poor visibility and greater potential for influence by interest groups.

Community‐based preventative interventions… are cross‐sectoral and there's no easy funding mechanisms for those now, and it's lobbyists and it's you know people that go to treasury… that are well organised and they'll, they might put in a business case or something … so anything that's kind of funded outside of those two formal mechanisms I think is, that's problematic.

Participant_14

The main challenges to integrating equity are mapped to potential solutions proposed by participants in Table 2.

**Table 2:** Challenges in integrating equity into HTA mapped to proposed solutions.

Identified challenge	Potential solutions
*Uncertainty*	
Public summary documents do not support improvement in the provision of equity information.	Include more detail within PSD's as to how equity influenced the decision (provides feedback to HTA actors)
Limited equity‐relevant data available	Encourage data collection, analysis and consideration of equity information pre‐HTA e.g. clinical trials, Therapeutic Goods Administration
Reliance upon qualitative equity evidence:	Equity‐informative economic evaluations can be submitted as an additional input within submissions where equity is a relevant concern
Limited HTA workforce capacity and capability to conduct evaluate and appraise equity‐informative economic evaluations	Investment in HTA workforce: additional training and funding of HTA evaluation activities to support inclusion and evaluation of equity
HTA actors are reluctant to adopt equity‐informative economic evaluations due to: Lack of awareness Unknown implications Fears it will add complexity to an already complex HTA system	Use case studies or pilots applying equity‐informative economic evaluations in relevant contexts to evaluate feasibility and applicability. Start with more socially acceptable equity categories to facilitate adoption.
	Include straightforward quantitative equity analyses (that do not require normative judgements on equity) in submissions such as: Subgroup analysis of efficacy, uptake, adherence etcetera (where possible) Distributions of disease burden or prevalence by social groups Extended‐Cost‐Effectiveness Analysis to explore equity impacts alongside economic evaluations.
Qualitative equity evidence is not well integrated into evaluation of technologies	Build skills and capability among HTA workforce to critically appraise and integrate qualitative evidence.
	Provide necessary resourcing of HTA evaluators to undertake integration of qualitative evidence
High‐quality, representative patient experience evidence is difficult to create due to limited transparency and short timeframes within the HTA process	Provide patient or consumer representative groups with more transparent information on upcoming technology appraisals and enable their engagement earlier in the HTA process.
	PICO process allows early patient/consumer input regarding relevant outcomes, include this process within PBAC pathway.
Stages prior to and beyond the listing of a technology also influence health equity but lack an equity lens	Revise HTA pathways ‐ apply an equity lens across all stages: Consider equity within safety and efficacy data Evaluate equity impacts of real‐world usage (uptake and effectiveness) Consider equity impacts within PBS/MBS restrictions Consider equity impacts within delisting or disinvestment decisions
	Establish an alternative pathway for evaluating technologies that address the needs of specific underserved populations such as Aboriginal and Torres Strait Islander people and children.
Equity must be considered in a consistently across all submissions for fairness of decision‐making, but the burden of including equity evaluations may be a disincentive to sponsors, potentially exacerbating inequity of access	Implement equity considerations into HTA strategically via a triage or early assessment stage (similar to PICO stage in MSAC) to determine when extensive equity evaluation is warranted. Consider and acknowledge potential for equity concerns in every instance. Request robust evaluation of equity impacts for technologies or contexts where equity impacts are significant.
Reactive HTA system where only technologies presented by sponsors can be appraised. Reliance upon sponsors to direct the types of technologies funded Other technologies may potentially reduce health inequalities lack a pathway for evaluation	Wide‐ranging HTA reform including: A single front door for all technologies (including digital, health or prevention programmes etc) Addition of a proactive arm where an equity‐focussed agenda targets healthcare funding alongside existing sponsor‐driven pathway.
Sponsors may face difficulties in meeting requirements for additional equity evidence, particularly where there are differing HTA requirements between countries.	Standardisation of the incorporation of equity across multiple HTA bodies to facilitate adoption through economies of scale.
*Ambiguity*	
Equity information does not enter HTA systematically as guidelines are open to interpretation	Clarification of HTA guidelines as to when and how equity should be included and evaluated
	Amend HTA guidelines to provide HTA evaluators and committee members greater agency to incorporate equity impacts.
Unclear directives to decision makers on how to consider equity within a deliberative process	Clarification of HTA guidelines as to how equity should impact the decision e.g. use of a framework that is applied to all decisions
Characteristics, beliefs and representativeness of HTA decision‐makers potentially influences decisions	
The lack of accepted societal equity priorities and preferences limits equity impact on decision making	Gather and incorporate public preferences and priorities on equity into HTA decision‐making: Mathematically e.g. preference studies/discrete choice experiments Deliberatively e.g. implementation of a Citizen's Jury
	Conduct further research on patient preferences for outcomes and ways of receiving healthcare.
	Further research to better define social groups, particularly with respect to intersectionality
Political risks associated with quantifying equity priorities for making redistributive equity decisions impede adoption of quantitative equity evaluations	PBAC and MSAC could establish and operate their own internal set of equity priorities (independence of PBAC and MSAC from direct government control)
In the absence of an accepted equity definition, the term ‘equity’ is employed by different actors to serve their own purposes creating confusion, frustration and tension.	Provide clarification in HTA guidelines regarding equity priority groups or categories, and definitions for the types of equity that will be considered in HTA.

## 5 Discussion

By developing a detailed understanding of how HTA actors include, assess and appraise equity, this study has explored a perceived gap between policy commitments and practice. Equity principles are a pillar of HTA in Australia [4, 39], however HTA actors discussed variability in how equity is incorporated and assessed, and how the challenges faced by decision‐makers limit their ability to integrate equity concerns.

Participants described HTA decision‐making as complex, nuanced and influenced by multiple inputs, with a wide range of equity‐relevant groups competing for attention. HTA is a complex policy community where decision‐makers face ‘bounded rationality' as they do not have the capacity to comprehensively gather, understand or give attention to all information relevant to their choices [40, 41]. In response, decision‐makers may instead rely on cognitive or procedural shortcuts [41, 42], and organisations may rely on standard operating procedures to help them ignore some information [40]. To handle this competition for limited attention, policy communities can seek to reduce uncertainty, through pursuing and translating more precise evidence, and to reduce ambiguity by adopting common interpretations or ideas to restrict the scope of policy problems [40, 43].

### 5.1 Reducing Ambiguity

This study found that unclear definitions of equity and a lack of accepted societal preferences for valuing equity create many of the challenges in integrating equity within HTA. These obstacles are well recognised and impede government policy action on equity more broadly [8, 44, 45].

Without a clear definition, we observed that HTA actors frame equity in different ways to serve their interests. Equity has proven difficult to define in Australian HTA with multiple concepts of equity described [46]. Diversity of equity concepts and the use of different frames by HTA actors can hinder understanding [47], and create ambiguity and confusion, leading to limited or fragmented policy action [48]. Strategic framing has been recognised within Australian HTA where access to innovative medicines was framed as an equity issue [49]. We have identified the potential ‘weaponisation' of equity where this strategic framing, within the adversarial HTA context, exacerbates tensions, potentially making conversations around equity challenging or combative, rather than constructive. Clearer HTA guidance may promote more constructive consideration of equity, but this may be difficult when government and HTA actors hold different definitions of equity [16].

HTA decision‐makers, or technocrats, face imperfect information on societal preferences for valuing equity. We found this disconnect between technocrats and the public limits the impact of equity. Inadequate and inconsistent inclusion of public values has been recognised as a major limitation of the Australian HTA system [50, 51]. HTA actors in our study perceived that public values regarding equity may not be well established or travel to technocrats through government directives (legislation, guidelines) as this is politically and ideologically challenging.

Societal preferences can be obtained through an aggregate approach such as stated preference studies and Australian equity weights have been elicited for some equity‐relevant variables using a discrete choice experiment [52]. Consistent with the literature, participants had concerns for the complexity, coherence, or transparency of weights, the potential for preferences to change over time, and the use of averages across a sample which may overlook differences or exclude outliers (whom may already be marginalised) [50, 53]. Alternatively, public values can be obtained through a deliberative approach. This involves reaching agreement on key outputs to inform policy through facilitated discussion among small groups, such as citizens' juries [50, 53]. Citizens' juries could help overcome the gap perceived by decision‐makers between research‐informed submissions and public preferences, but these can be costly, and may struggle to capture representative, unbiased and diverse perspectives [53, 54]. A framework integrating aggregate and deliberative approaches may better provide decision‐makers with necessary information on public values [53].

### 5.2 Reducing Uncertainty

Decision‐makers are provided insufficient information to meaningfully integrate equity. We described how limitations pre‐HTA and in national data collections limits capacity to generate equity evidence. National data custodianship that protects and pursues equity considerations is needed [55]. Furthermore we learned that equity‐relevant information is inconsistently integrated into submissions and assessment. Patient engagement has been shown to increase attention to equity considerations in HTA [56]. Despite recent changes in HTA policy [27], we identified that challenges in creating high‐quality patient experience evidence due to resource, time and transparency constraints of the HTA process persist [57]. Patient or consumer evidence was perceived as inadequate and not representative. Public and patient participation in HTA is known to be suboptimal, with underserved groups facing barriers to meaningfully participate, and funding decisions favouring those with more power and resources [58, 59, 60]. Providing clearer criteria for selecting stakeholders and earlier engagement of patient or consumer stakeholders can enable their contribution to the assessment stage [56, 58, 60].

Conflict over what counts as evidence between health professionals or advisory committee members and sponsors, public and patients has been identified [57, 60]. Despite broadening of PBAC/MSAC committee membership over time [27], we revealed concerns that decision‐makers, particularly clinicians follow an evidence‐based biomedical paradigm that de‐prioritises real‐world or patient experience data. However, HTA experts may prefer to focus on rigorous, unbiased evidence, because their perceived role is to be objective and apolitical [61, 62].

Within this technocratic approach, ‘lack of data' is viewed as a problem, prompting decision‐makers to seek more evidence to reduce uncertainty [63, 64]. As in the United Kingdom, we found concerns with data limitations, uncertainty and complexity impede the adoption of EIEEs [65] It has been argued that uncertainty, insufficient standards of evidence and scientific complexity can be used to justify policy inaction [64]. HTA appraisal committees must acknowledge the conflict faced between these opposing epistemic approaches to knowledge and support an institutional culture that values both perspectives [60].

### 5.3 Policy Implications

This study furthers our understanding of how equity is operationalised within Australian HTA in many ways. It is well established that deliberative processes are valued within the Australian HTA context [2, 27]. We found this opinion extends to all HTA actors and identified widespread support for the continued use of qualitative equity inputs. We provide a detailed explanation for these preferences as well as signposting potential pathways for incorporating quantitative equity analysis such as ECEA or small DCEA pilots. This understanding of stakeholder preferences with respect to EIEE's is crucial to their adoption.

Consistent with the literature, we found the challenge with these deliberative processes is that they are informal, unstructured, and unstandardised, allowing the behaviour and personal values of individuals to influence how these ‘less quantifiable factors' are considered [66, 67]. These issues can be addressed through transparent frameworks and improving stakeholder participation [66]. Proposed frameworks include the Assessing Cost–Effectiveness framework (cost‐effectiveness (or utility) analysis combined with explicit discussion of ‘second‐stage' criteria, including equity) [46, 68], MCDA (consideration of a pre‐specified, ranked set of criteria) [67], and ‘evidence‐informed deliberative processes' (which combines A4R with MCDA) [66, 69]. Successful integration of such new approaches into policy may require support from researchers or experts and advocacy from powerful stakeholders [70]. Deliberative process can also be improved through stakeholder participation in deliberations. Including the right voices, such as affected patients and relevant clinicians, and ensuring all stakeholders have ‘equal opportunity to influence the outcome' are important to the consideration of equity impacts [68, 71].

Notably this research looked beyond decision‐making to reveal how pre‐appraisal stages can also impede the systematic consideration of equity, where integration depends on the characteristics, experiences and agency of individuals. Clearer HTA guidelines that define when and how equity should be included and assessed would help make equity ‘everyone's business but still someone's responsibility.' [48] Similarly, providing the rationale for decisions in PSDs benefits the public through improved transparency [27], but we found this is also important to improve the equity information provided in submissions.

By identifying the conditions when equity is most impactful, we have located strategies that can facilitate its consideration. Our findings corroborate that equity influences vaccine funding decisions for Aboriginal and Torres Strait Islander peoples [21], and attribute this to several factors: a social group that is well‐defined and seen as deserving of distributive action, inclusion of quantitative equity analysis, use of evaluative criteria which explicitly include equity, funding structures that enable differential listing by patient subgroups and decisions with limited budget impact. We have linked the challenges to integrating equity identified in this study to strategies proposed by participants strategies (Table 2).

HTA actors felt equity should be better considered at all HTA stages not just appraisal. The equity checklist for HTA (ECHTA) could partly fulfil this need, providing guidance on equity considerations for five HTA phases: scoping, evaluation, recommendation, implementation and reassessment of decisions [72]. Future efforts to expand such a checklist, spanning from the creation of safety and efficacy data to disinvestment, would align with the objectives of HTA actors in our study. Additionally, participants described both the need to consider equity in all submissions for fairness, and feasibility concerns with incorporating and evaluating more extensive equity information. We found support for an approach combining consistent attention to equity impacts in every submission with the use of a triaging approach at the scoping stage to identify submissions where a more comprehensive equity evaluation is warranted. The ECHTA checklist provides such flexibility where the extent of equity consideration can vary on a case‐by‐case basis depending on the context [72].

Governments and HTA agencies must be explicit about which equity concerns are of most interest, particularly when conducting DCEA [23, 26]. Equity concerns can be for those with a particular disease versus the general population, for specific social groups such as PROGRESS‐PLUS or by the receipt of (or access to) healthcare [26]. We have found HTA actors hold concerns across all these dimensions with no consensus. Priorities were seen as dependent on the current socio‐political climate, therefore establishing equity priorities is a critical but difficult challenge.

Lastly, the literature describes a tension between procedural fairness (decision‐making) and substantive fairness (the decision itself, including distributive equity) [27, 73]. Our study identified this tension within the conflicting equity frames used by HTA actors. Better consumer involvement and consideration of context may help resolve this tension [27]. Instead of viewing these as conflicting goals, we should strive to achieve both; transparent and fair processes that systematically include distributive equity criteria. The Evidence and Value: Impact on Decision Making framework and ‘evidence‐informed deliberative processes' have combined structured analysis of evidence‐based components (MCDA approaches) with deliberative consideration of ethical elements [69, 74], to provide both procedural and substantive fairness. The development and implementation of an HTA framework that meets the needs of stakeholders, meaningfully incorporates health equity and is feasible, remains elusive.

### 5.4 Strengths and Limitations

Adopting a qualitative approach, including multiple perspectives, enabled creation of a nuanced understanding of the HTA decision‐making context [75]. Our findings relate to Australian HTA and may have limited transferability to other contexts [76]. However the PBAC is seen as influential internationally, acting as a potential driver for global change [77]. Our findings should be interpreted as participants' personal opinions, not as representative views of the participants' committees or organisations.

The study was limited to Federal‐level HTA because the funding and operational context for state and territory decision‐making is complex and heterogenous, and perceptions of fairness differ among actors at different levels of decision‐making [78, 79]. Resource allocation decisions occurring at the state level were however seen as relevant to reducing health inequalities, providing opportunities for future research. It is also important to understand public perceptions of the HTA process, however this was not explored this study. Finally, this study reflects the present, but long‐term policy analysis pre and post implementation of the HTA review recommendations, applying policy theory such as punctuated equilibrium, would be valuable to study the dynamics of policy stability and change [80].

## 6 Conclusion

This study identifies institutional, methodological and conceptual challenges relevant to the systematic and impactful integration of equity into HTA. Greater clarity within HTA guidance, in the conceptualisation of equity, and in the articulation of public values and equity priorities is needed. In the context of forthcoming HTA reforms, this analysis may provide useful insights to inform the ongoing evolution of equity consideration within Australian HTA.

## Author Contributions

**Sheridan Rodda:** conceptualisation, investigation, writing – original draft, methodology, writing – review and editing, formal analysis, project administration, visualisation. **Melanie Lloyd:** conceptualisation, methodology, validation, writing – review and editing, supervision. **Aili Langford:** conceptualisation, methodology, validation, writing – review and editing. **Jedidiah Morton:** conceptualisation, writing – review and editing, supervision. **Zanfina Ademi:** conceptualisation, methodology, validation, supervision, writing – review and editing, funding acquisition.

## Conflicts of Interest

Zanfina Ademi is a member of the ESC of the PBAC. There are no other declarations of interest.

## Supporting information


Supporting File 1

Supporting File 2

Supporting File 3

Supporting File 4

## Data Availability

Due to the sensitive and confidential nature of the data collected in this study, the data are not publicly available due to privacy or ethical restrictions.
