# Supplementary material for: How Is Equity Considered Within National Healthcare Priority Setting? A Qualitative Study of Australian Health Technology Assessment Actors' Perspectives
Source: Health Expect. 2026 Oct 30;29(6):e70955. doi: 10.1111/hex.70955 (PMC13722783; doi:10.1111/hex.70955)
Supplement: Supplementary file 1 [file HEX-29-e70955-s001.docx]

**Supplementary File S2: Methodology**

Further detail on study methodology is provided below.

*Epistemological and ontological approach*

We adopted a critical realist perspective with a contextualist epistemology was adopted for this stud to acknowledge that reality can only be partially accessed, as it is shaped by how it is experienced and interpreted by individuals within their social context.[1, 2] Knowledge is seen as provisional and context-dependent, and is produced subjectively.[1]

*Sampling and sample size*

Purposive sampling with snowballing was used to ensure adequate participant diversity. Participants were identified via publicly available information and recruited via email. At interview conclusion, participants could suggest other potentially eligible contacts. We applied the concept of information power [3] throughout data collection to evaluate sample size across five dimensions as follows:

- Study aim: our study had a broad exploratory aim
- Sample specificity: the intention was to garner perspectives across diverse participant groups
- Application of theory: theory was not instrumental in the study design
- Analysis strategy: the analysis did not involve cross-case comparisons
- Quality of dialogue: the dialogue in interviews was strong and detailed

*Data generation*

The interview guide was pilot tested to ascertain whether the content could be adequately covered in the desired timeframe and to ensure there was no fundamental misunderstanding or confusion in the definitions and content of the guide. The guide was piloted once only as minimal issues were encountered with interpretation of the content or scope, and depth or length of discussion.

Pilot testing was conducted with a health economist familiar with equity concepts and the Australian HTA context. This person was chosen as they were representative of the majority of intended study participants: whom would have either health economics training (most HTA committee members, HTA evaluators, academics, industry), or at a minimum, some exposure to health economics concepts through their recruited role (some HTA committee members, patient representatives and NGO participants).

The interviews were transcribed verbatim using the Whisper automatic speech recognition program* that was run locally on SRs computer for data security and confidentiality purposes. These outputs were reviewed, checked and corrected where necessary by SR to create the finalised transcripts for analysis. To help with readability:

- ellipses were used to omit informal or repetitive phrases that did not impact the meaning of the content e.g. ‘ah’ ‘um’ ‘you know’
- words contained in square brackets were used where necessary to clarify meaning
- words in curved brackets were used to indicate non-verbal information such as laughing or thoughtful pauses.

* Open AI™ https://openai.com/index/whisper/”

*Analysis*

In brief, SR and ML familiarised themselves with the dataset then coding was conducted by SR. The analysis was predominantly inductive with coding grounded in the data, prioritising participants’ experiences and perspectives viewed through their context.[1, 4] Codes were reviewed and refined before a second round of coding. SR constructed initial themes from the finalised codes. Themes were reviewed by ML to ensure they adequately reflected the dataset then refined by the research team (SR, ML, AL, JM, ZA) to ensure the themes were coherent.

We did consider whether to evaluate themes and perceptions across broad participant categories. Cross-case analysis was not conducted:

- because upon engagement with the dataset, we did not identify meaningful and systematic patterns across participant groups or types.
- to maintain confidentiality for study participants as the HTA community in Australia is small.

Where minor differences across participant groups or roles were recognised, these were used in theme construction and provided in the Results.

We considered the potential to apply policy theory. As the aim of the project was exploratory, theory was not applied to data collection or the data analysis a priori. However, in reflexive TA, themes are created at the intersection of data, researcher’s subjectivity, and their analytic process, therefore the analysts existing knowledge of health policy theory influenced theme construction. [4]

*Reflexivity*

We acknowledge that the personal and cultural perspective of the researchers is integral to how data was collected and analysed in this contextualist study [1] and the ‘positionality’ of researcher influences the way policy studies are conducted.[5] Our research team comprised a diversity of genders, career experience and backgrounds (health practitioners, health economists). We have an interest in the integration of health equity into HTA and possess the power and resources provided by academia to pursue this research interest. Nonetheless, we approached study design and analysis with genuine curiosity and a desire to generate new knowledge irrespective of whether this supported our research agenda. Further, a reflexive journal was kept by SR during data collection and analysis to maintain critical awareness of her interactions with participants and with the data. In terms of positionality, the research team included both policy process ‘insiders’ whom can access a stronger, more authentic but potentially biased understanding of the culture or phenomenon being studied, and ‘outsiders’ whom are unfamiliar with the context, can achieve greater curiosity, and raise alternative viewpoints.[6] Combining both insiders and outsiders may yield a richer, comprehensive understanding of a policy process.[5]

References:

1.	Madill A, Jordan A, Shirley C. Objectivity and reliability in qualitative analysis: Realist, contextualist and radical constructionist epistemologies. The British journal of psychology. 2000;91(1):1–20.

2.	Clarke V, Braun V. Successful Qualitative Research: A Practical Guide for Beginners: SAGE Publications; 2013.

3.	Malterud K, Siersma VD, Guassora AD. Sample Size in Qualitative Interview Studies:Guided by Information Power. Qual Health Res. 2016;26(13):1753–60.

4.	Braun V, Clarke V. One size fits all? What counts as quality practice in (reflexive) thematic analysis? Qualitative Research in Psychology. 2021 2021/07/03;18(3):328–52.

5.	Walt G, Shiffman J, Schneider H, Murray SF, Brugha R, Gilson L. 'Doing' health policy analysis: methodological and conceptual reflections and challenges. Health Policy Plan. 2008 Sep;23(5):308–17.

6.	Meusel V, Mentzakis E, Baji P, Fiorentini G, Paolucci F. Priority setting in the German healthcare system: results from a discrete choice experiment. Int J Health Econ Manag. 2023 Sep;23(3):411–31.
