# Supplementary material for: How Is Equity Considered Within National Healthcare Priority Setting? A Qualitative Study of Australian Health Technology Assessment Actors' Perspectives
Source: Health Expect. 2026 Oct 30;29(6):e70955. doi: 10.1111/hex.70955 (PMC13722783; doi:10.1111/hex.70955)
Supplement: Supplementary file 2 [file HEX-29-e70955-s002.docx]

Supplementary file S4: Themes and subthemes with supporting quotes

**Theme 1: Equity impact on decision-making is limited and not transparent**

| **Brief description** | **Supporting quotes** |
| --- | --- |
| Despite existing legislation and HTA guidelines, decision-makers can let equity concerns meaningfully impact their decision – there some degree of freedom.  Equity-informative economic evaluations are permitted to be submitted to PBAC or MSAC as a supplementary input.  Equity issues are regularly raised or acknowledged within the decision-making stage, particularly for Aboriginal and Torres Strait Islander people, rural and remote communities, rare diseases or high unmet clinical need and children.  Socioeconomic status may be assumed to be already addressed through universal subsidy (PBS/MBS). Socioeconomic inequalities may be better addressed beyond HTA.  The impact of equity on decisions was perceived to be modest in most instances, with cost-effectiveness the dominant input.  Equity concerns can be relevant (successfully or unsuccessfully) to pricing negotiations post-recommendation.  The role of equity within the decision-making process is opaque.  This poor transparency is seen to be due to the deliberative process with multiple inputs. Factors that may directly or indirectly influence the deliberative process that include: patient or consumer groups, public or consumer comments, clinicians, the government agenda, budget impact, precedence and legislation.  The multifactorial deliberative process can leave decision-makers open to external influences (industry, interest groups, advocacy groups etc). For example, industry can potentially influence decision makers via patient or consumer representative groups or government stakeholder engagement.  Disease-specific advocacy groups that are organized and more well-resourced (the ‘loudest voices’) receive more attention and consequently more funding.  These groups can use arguments for ‘equity of access to treatments’ to support their advocacy, though their definition of equity may not be consistent with that which is considered socially determined, avoidable or unfair.  Participants were concerned that underrepresented or disadvantaged populations have less capacity to advocate for themselves which is inequitable. | “There is a legislative and statutory framework for PBAC decision-making. There isn't one for MSAC, right? …. PBAC can't recommend a drug which is more expensive unless it accepts that it is more effective than existing therapies, with the qualifier and it's an important qualifier, for at least some people. So it doesn't have to be for every group. So that's the basis under which those sorts of decisions can be made for one group and not another group in a legal sense within the PBAC arrangements.” Participant 15  “you've got your base case, which should be very much guideline driven. And then if you feel there's really special, you know, there's other special considerations that need to be considered, for example, the inclusion of productivity impacts or the inclusion of carer costs, or it could be a distributional cost effectiveness analysis. That's where that should be done.” Participant 14  “Well, I would say at every meeting discussions of equity are raised, but they are very much that qualitative aspect. So, they're usually a discussion of how's this going to affect rural populations? How's this going to be different for, I mean, the rural urban thing is the one that comes up the most.” Participant 8  “they acknowledge that it's important. You know, again, if your drug’s not cost effective, (laughs) I don't think it's going to change the assessment.” Participant 6  “I have very limited experience of anybody discussing more like an income distribution type related in equity, a rich-poor distribution. I mean people will talk about the out-of-pocket cost barriers, um but they…the conversation doesn't go down in inequalities or inequities. You know, people can talk about out of pocket costs as a barrier without necessarily thinking about that as an unfair inequality.” Participant 8 |
|  | “The system that I've experienced is pretty well designed to give as many opportunities as possible to raise additional information such as distributional information. But equally, it's quite rare to see ahh (pause} economic evaluations where the ICERs are reported separately for instance, different deciles of income or regional and remote versus metropolitan…” Participant 12  “a case study is xxxx where they tried to focus from an equity perspective on the…and it was rejected by PBAC quite emphatically, I think. ….the fiscal impact was significant, and so the equity essentially… weighted inherently didn't overcome the total financial impact of that submission, and it was unsuccessful…despite it being grossly aimed towards improving equity.” Participant 21  “I get the feeling that in general the department [of health] is supportive and also finds it useful to have an equity issue well defined or documented so they can use that in negotiations with the government…” Participant 5  “So we include them [equity information] because they need to be brought to the attention of the HTA, but we never get a sense of, um you know how much it factors into the decision making. That's a bit of a black box.” Participant 4  “the risk there is that… if you can't quantify it, then you… the committee might be making decisions which aren't transparent and consistent. One of the great advantages of the cost per QALY metric is that it is transparent. You can see how it's built, you can replicate decisions, and even if people will disagree they'll understand why you've made the decision.” Participant 12  “the decision-making process for both committees involves a lot more inputs other than the health technology assessment…that includes the opportunities for consumers, and patients to provide inputs, for clinicians to provide inputs….That includes advice from government that says we'd like to see, you know, we would like to see this put in place for some reason.” Participant 15  “So we pushed pretty hard… a couple of years ago with PBAC… to list things and to consider equity for xxxx more wholesomely. And it worked for a while, but I think…when you're reliant on the goodwill of committee members to do this, you can burn your capital with relationships, if you push too hard. I think this is part of the issue when you have a more vague way of managing this at a personal level. That, um, and it's not an indictment on any one person… it's more about the system.” Participant 21  “a small patient charity, which was heavily pharma funded… were sort of… I guess used in a way to be a patient voice for equity concerns and a more credible voice for, and a credible sort of trusted voice to government on those…” Participant 17  “I'm actually really concerned around people that aren't on concession cards. They're those low income, you know, families that just above that threshold who aren't getting a lot of those concessions and I think are hit hard. They're very hard to identify, not necessarily in the data, but as a community. Who advocates for them? Who represents them on consumer groups?” Participant 16  “the state of the way things are in xxxxx funding, both for research and for care, is that the louder voices, the people who are more articulate and are better organized and are in that sort of higher SES group who have greater kind of, societal kind of connections are able to (laughs) you know, they're able to influence things a lot more.” Participant 17  “some groups or treatments or whatever have access to a lot… to a number of treatments and support. I mean, classic example would be like breast cancer, right? Like you've got breast cancer, you know, you've got lots of support around you, great organizations in that space, great… advocacy…whereas…you deal with another type of cancer, uh, yeah, big toe cancer or something. And you just like, well, there's just no one advocating for you. Um, and, and, and so I guess I see it in, yeah, I guess the access to treatments, but then also the support structure in the advocacy networks that sit around that.” Participant 13  “one of the particular…cancers, which is unbelievably kind of high burden is liver cancer… it receives very little attention, but predominantly because the groups that are affected by liver cancer are like Chinese-speaking men, intravenous drug users, you know, people with a history of alcoholism. It's really marginalized people. So I guess really what I'm trying to say here, is that… the groups that receive the most funding are the groups that have the most powerful and articulate advocates for them.” Participant 17 |

**Theme 2: Institutional, methodological and ideological challenges impede the consideration of equity**

| **Subtheme** | **Brief description** | **Supporting quotes** |
| --- | --- | --- |
| 2.1 Equity information enters the HTA process inconsistently | Equity enters the HTA process through multiple routes in a context-specific manner. Equity information can be included by sponsors (or economists building submissions), HTA evaluators, academics, patient or consumer representatives, HTA committee members and decision-makers (MSAC or PBAC members).  There are contrasting opinions on whose responsibility it is to raise or include equity concerns. When actors think equity rests with someone else, it can be seen as no-one’s responsibility.  Insufficient clarity in the HTA guidelines allows the inclusion, assessment and consideration of equity to be open to interpretation by all actors. The incorporation of equity is influenced by the beliefs, awareness, experience and work context of individuals.  There were conflicting views among HTA evaluators on the incorporation of equity. Seen as both their role to bring attention to equity versus maintaining a focus on safety, effectiveness and cost-effectiveness. Evaluators were willing to include equity analyses if this was requested and they were resourced to do so.  Dismissal of prior efforts to submit equity information creates frustration and apathy towards including equity concerns in future submissions.  Decision-makers were seen to have agency to consider equity, but actors prior to the decision-making stage, may not feel empowered to incorporate equity information.  Individual aspects (bias or ideological views) of decision-makers were perceived (by non-decision makers) to influence the consideration of equity. Due to the nature of their role, decision-makers may not be representative of the public and this was perceived to impact equity consideration, particularly the predominance of clinician members.  Clinician members were perceived to potentially hold certain perspectives such as a focus on access to innovative treatments and limited capacity to engage with distributional equity or equity-informed economic evaluations.  Public summary documents don’t provide detailed feedback on what aspects of the submitted equity information was useful or how it impacted the decision. Therefore, sponsors may be disinclined to produce equity information and all HTA process actors cannot improve their provision or assessment of equity information to meet decision-maker needs | “It just creeps in and it's very dependent. You know, so I mean, I imagine you could have the same submission evaluated by three different sponsors and mostly economics might look pretty similar…The things that get brought in like equity, I think would be very dependent on who looked at it and possibly even who within that evaluation team” Participant 5  “Consumer advocates are very good at speaking up about equity, and always have equity I think at a higher level in their mind than I think a lot of us who are doing the clinical and economic aspects do, necessarily. So they're sort of like if you like the guardian of equity in the meetings um, and in the system.” Participant 5  “We just do it because we are public health people and that is something that is of interest to us.” Participant 9  “So even though it's not asked, wasn't specifically asked for by government, we decided that to do a proper HTA, you need to incorporate that [equity-relevant information].” Participant 9  “at my stage, it's probably only just, you know, signposting there's this problem, potentially, revive what's been already provided the submission, maybe a little bit of extra context, but it's not, you know, our role in the process is mostly to flesh out the science and to make sure the science is solid.” Participant 2  “ultimately the PBAC is the service, I’m a service provider, in which case if they want me to do something I will do it. [laughs] …as soon as there's… sort of like analysis to happen, and for us to include something like, sure, not a problem.” Participant 19  “so the, the hold up I guess from your perspective is just that…there's just no comment made under whether it should or shouldn't be there?” Interviewer “Yeah, there's not standard, it's not part of our contracts to do this. It's not funded as such.” Participant 9  “I can recall one very specific example when we were making a submission to the PBAC where we actually went out and collected qualitative data on the health condition, …. and that, that information was completely disregarded by the reimbursement agency.” Participant 18  “We're very careful that what we do is developing the evaluation for MSAC to consider….We're just saying, does this do the job it's meant to do? And is it fit for purpose for decision making? And so, if there was a guideline that said, and these are the equity things that you do, then we would be able to say that. We'd be able to say, oh, there's not enough consideration of equity in this analysis. Um…  “But you need that guidance.”  “It needs to go in that sort of guideline.” Participant 8  “whether or not they want to *add* to that list of items in the guidelines that's up to them… it's up to them they just tell us what to do.” Participant 19  “it's left to the Department of Health and a select member of individuals on a committee. Which may or may not be representative of the Australian population.” Participant 19  “when you think about the people who are sitting around that table in the committee, they're wealthy, well-educated people who won't necessarily have a socialist mindset um, about health care.” Participant 8  “because at the moment I think it is kind of like all of that decision-making is done by clinicians and, you know, although I think the clinician voice is important, I don't think it should be the only voice we're hearing from when we're making these decisions.” Participant 13  “I think because the people who sit on the committees are coming from, well, certainly the PBAC committee, are almost all clinicians. And I think clinicians are often thinking about equity, but on a one-on-one patient basis much more than in a global, you know, in a bigger scale and understanding in a bigger scale, you know,” Participant 5  “I would say that largely that's because nobody else um, on the xxxxxx committee, nobody else would know what it is. They wouldn't think in distributional terms, they wouldn't think very often in equity terms, in terms of an analysis. They're all clinical discussants…”  “I mean, feedback is minimal. Like we, we do get like bits in the public summary documents sometimes saying like, Oh, this was kind of like useful…So yeah, the feedback mechanism is really lacking….They just like write some kind of essay about like all the stuff that, you know, all the uncertainties and it's just like this diatribe of stuff, but it doesn't actually help you to really, you know, figure out a way to address the issues.” Participant 13 |
| 2.2. Insufficient equity evidence provided to decision-makers | Decision-makers are presented with limited equity evidence.  Patient or consumer evidence is seen as crucial to considering equity impacts. Better quality patient experience evidence is wanted by all actors.  However patient or consumer evidence is perceived as inadequate and not representative, with concern for the exclusion of underserved groups. Some patients or conditions have greater capacity to participate than others, potentially entrenching inequalities.  Groups potentially affected (as described by participants) were:   - Aboriginal and Torres Strait islander people (separate committee representation comes from the National Aboriginal community controlled health organization) - culturally and linguistically diverse groups - younger people - those with limited internet access - people of low socioeconomic status - people with mental illness   Creating high quality patient experience evidence is challenging because: there is limited guidance, it is resource intensive, there is limited transparency of upcoming agendas, and organizations face short timeframes. Therefore, support is often needed from umbrella patient advocacy groups or the government Consumer Evidence and Engagement Unit.  Enabling patient engagement earlier in the HTA process would provide patient or consumer organizations more time and improve the patient evidence submitted. | “I just don't think that there's, there's evidence coming through to, to actually support equity most of the time. So if you want to increase equity in your health system, I'm not sure the evidence that comes into HTA prior to appraisal actually helps you to do that. And HTA needs both.” Participant 7  “The first is the information that's available. It's surprising how little we do sometimes know about distributional elements of some of the conditions that we look at. A lot of assumptions are made about access, which are not necessarily correct in terms of how people access things…So having …evidence… an informational base... So having an informational base for equity.” Participant 15  “a lot of our submission work these days would include, we try to, in all cases, suggest that sponsors include the patient voice in the submissions and that lived experience piece, because that is so important in trying to highlight you know are there equity issues…” Participant 13  “obviously, no one can have everyone's experience, but it would be better if we had some more data to sort of guide us… And that's where I think the consumer liaison can help because they're collecting that information, at a more ground level. But then again, that's always incomplete. It depends on who participates, who writes in for the consumer experience, and they don't go out and actively seek opinions from groups that may be affected, for example. So I think that's the biggest barrier really.” Participant 5  “we have had trouble getting into CALD communities. We have NACCHO on our advisory committee, but we don't have First Nations experience. We would feel that we were being pretty inappropriate if we just wandered off ourselves and tried to do that. So we work with NACCHO on those sorts of things… our networks are pretty deep after so many years, but new communities spring up for the time and um, and one of the hardest things is Patient organizations are not hard to find, but younger people don't tend to join patient organizations the way they used to. And that's why our online work is so important… we don't necessarily ever hear back from people… not everyone has access to the internet, of course, but that's a huge amount of people in there who aren't being heard in other ways.” Participant 7  ““I'm actually really concerned around people that aren't on concession cards. They're those low income, you know, families that just above that threshold who aren't getting a lot of those concessions and I think are hit hard. They're very hard to identify, not necessarily in the data, but as a community. Who advocates for them? Who represents them on consumer groups?” Participant 16  “And people with severe mental disorders…they're not dying of suicide, which is what a lot of people think. They're actually dying from chronic disease that no one seems to care about. Right…and they don't lobby…They're not like some groups you know who are really active lobbyists and you know get out there and they've got they're very polished and they're very, you know whatever. There are you know mental health NGOs and things like that but, I don't know, they're just…. I think some groups are just more stigmatized than other groups.” Participant 14  “It's a very challenging space. I mean, you know, trying to collect this sort of data and put it in a way that's kind of meaningful to decision makers is always an ongoing challenge. And I think there's kind of like a lack of methods in that space to really do that... it's not kind of possible to go and survey the whole patient population and timeframes that you have. So you sort of go and do something that works.” Participant 13  “Trying to find people is a real difficulty... So we have had trouble getting into CALD communities…and one of the hardest things is Patient organizations are not hard to find, but younger people don't tend to join patient organizations the way they used to. And that's why our online work is so important…” Participant 7  “working in a role with, between department and company on providing a summary of information for patients so that they actually, the organization actually knew what was in the application. So they could give input, which is a slightly funny thing we have where they can't always see what's in the application they're trying to give comment on.” Participant 7  “you know the time challenge would be avoided if patient organizations were involved much earlier in the process. So I think there are process issues there… unless someone tells us, we're not aware until we actually see the agenda of what's being considered. So it'd be really great to be able to share real world data and information at an earlier stage of this process.” Participant 10 |
| 2.3 What counts as evidence? | The HTA guidelines prescribe what types of evidence are to be included for consideration. Decision-makers firmly follow the guidelines (excluding evidence where indicated), but some feel constrained by them.  The deliberative stage can consider multiple informational inputs of various evidence types, however decision-makers are seen to prefer high-quality, quantitative evidence such as randomised controlled trials (RCTs) and the base economic evaluation, giving less weight to other types of evidence (qualitative, patient or lived experience etc) that is often used to support equity impacts.  The high proportion of clinician committee members was discussed, and clinicians were perceived to strongly prefer RCT evidence following a biomedical paradigm of health based on evidence-based medicine rather than serving the interests of the system (the public or taxpayer).  Decision-makers may choose to disregard some types of evidence such as equity-informed economic analyses if they do not know how to appraise them.  There are conflicting views as to what types of evidence should be considered in HTA. Decision-makers and government hold this more restrictive view while sponsors, interest groups and patient or consumer groups believe serious consideration should also be given to other types of evidence as well such as real-world data, qualitative studies and patient narratives. For example real-world data on equity impacts was useful during the COVID-19 pandemic.  The choice as to what constitutes evidence in HTA can have implications on the type of research funded and conducted in the future. For instance, a focus on RCT data for curative treatments, especially cancer, may perpetuate more of these types of treatments while other therapeutic options (eg for women or children) remain underexplored. De-prioritisation of qualitative evidence in HTA may result in less research on patient preferences or experiences being conducted.  What’s measured can be acted upon. Currently national datasets do not provide sufficient equity-relevant information to support equity-informed analyses. Government choices about the type of data we collect may impair our ability to consider equity. | “we have to work within the confines of what is required of the… the guidance in the guidelines… Now, the guidelines clearly say no carer QALYs, right? And they're in there, so we just ignore those, we take them out. I'm not saying I personally agree or disagree with that, but right now we have to follow the process and follow the guidelines…” HTA6  “going back to rare disease…in some ways the input from the community is very valuable, but then it also plays against the likelihood of the trial design being something that's less than the PBAC wants to see, as in, you know, it's not double blind randomized, it's historical control data. So I made a submission to the government using a historical control for what was a rare disease and… it blew their minds. They couldn't consider it and we had to make at least three submissions to have that medicine approved…we probably spent more on decision making across companies and the agencies involved and… than we did on what it would cost the community…” Participant 18  “I think because the people who sit on the committees are coming from, well, certainly the PBAC committee, are almost all clinicians.” Participant 5  “ because evidence-based medicine is all randomized studies and it's hard for us to present anything to clinicians that is not randomized, because they don't, they can't conceive non-randomized evidence as being worthy or of at the same level.” Participant 4  “So health technology assessment was established from perhaps a medical scientific perspective. And then health economics in the 90s came into play a major role… and it's established to support the health system. But that health system only exists for the sake of us when we need health care. So we try to look at what it would be from a patient perspective. And by that, we mean multiple patients and carers and those who engage with the health system.” Participant 7  “if somebody presents something new, somebody’s got to be able to evaluate it. (laughs) And so if nobody on the committee goes, has any experience with it, then it’s likely to be discounted. Yeah, because you can’t interpret it.” Participant 15  “It's not only interpreting, it's valuing it to the same level as they value… the clinical evidence coming from randomized studies. I see it every day in HTA, you know, you give them a randomized study, but you support it with real world evidence. And mostly they ignore the real world evidence because they say only a randomized study has the elimination of bias, which is not necessarily correct… we've brought into their stats and their language as economists because we've had to.” Participant 4  “in the COVID antiviral space, for example, were able to use real world data to support PBAC making a decision, that targeted Aboriginal and Torres Strait Islander people to improve accessibility of those antiviral drugs… And that worked quite well… the government were willing to look at real world data to, in my opinion, improve the equity of a decision, and target a specific population.” Participant 21  “I would like us to better use qualitative and quantitative research that captures patients’ needs, preferences, experiences and perspectives as being fundamental to the evidence base” Participant 7  “when you talk to industry people, I think they often focus on rare diseases or rare cancers… I think that's the, that's how R and D sort of pipelines are evolving. And, you know, you increasingly get more targeted and targeted therapies… Again, I think that's why it's, it's overinflated is cause that's the medicines that are available.” Participant 6  “I don't think the equity issue actually sits with the funding body. I think the access sits with uh, with what people are bringing forward… And I think another area which I haven't touched on is the issue around gender. And again, that's a specific issue because it's in part about what medicines have actually been presented” Participant 15  “What gets measured is often what gets assessed. And, you know, we may not have data around every priority group” Participant 6  “in a world where you can't accurately measure distributional impacts of an intervention, there's a tendency in certain settings for them to be of diminished importance because what you can measure becomes important.” Participant 12  “I think that you don't have the data to do it properly. And I think, you know, if you need the Australian Institute of Health and Welfare and the ABS to actually start producing the right data that can be used. So at the moment, I don't think we're there yet.” Participant 4 |
| 2.4 Equity is weaponised | The Australian HTA system is seen as reactive, responding to submissions rather than setting an agenda that includes equity goals. This reactive nature is perceived to limit our ability to reduce health inequalities.  The HTA process is seen as a barrier to access to technologies. Also, this reliance upon the sponsor for some types of data restricts capacity to evaluate equity impacts.  The HTA process is adversarial and ‘gaming the system’ occurs where sponsors utilise existing rules, guidelines and thresholds to tailor submissions to their advantage. This is recognised by the government and decision-makers, creating a context of suspicion and tension. The adoption of more structured equity considerations into HTA guidance is seen as risky due to concerns for ‘gaming the system.’  Equity is a complex term with multiple interpretations. For instance, participants held equity concerns across a wide variety of social groups with no consensus as to which should be prioritised. Social groups for which participants held equity concerns included: Age (children and older people), Aboriginal and Torres Strait islander populations, culturally and linguistically diverse populations, carers, physical and mental disability, sex, geographical location, rare disease, socioeconomic status, unmet clinical need.  In the absence of clear definition of equity in HTA (for instance within HTA guidance or other government directives), actors can frame ‘equity’ in self-serving ways. Four definitions or ideas of equity were described:  1. ‘Equity of access to innovative treatments’ to compensate for unfair future QALY losses due to disease severity, rarity, childhood or high unmet clinical need is a dominant frame. This was a dominant framing, commonly used by sponsors, clinicians or patient/consumer representatives that was present all throughout the HTA process.  2. Decision-makers discuss ‘Equity of access to care’ giving consideration to treatment pathways to ensure equal access to publicly funded technologies.  3. ‘Social equity’ aiming to compensate for baseline disparities in health across social groups is the least dominant frame, mostly used by academics, economists, evaluators and some decision makers.  4. Decision-makers raise ‘equity of decision-making’ representing a fair and consistent decision-making process that does not unintentionally make some worse off.  Within the reactive HTA context, this framing of equity can create tension and frustration. Within the social norm that equity is undeniably important, the term ‘equity’ can be weaponised - used as a device to support a proposed argument or position.  Equity narratives may be used to highlight the impact or benefits of a technology eg. ‘equity of access’ for rare diseases being used to endorse funding of specialised treatments for small diagnostic subgroups, or clinicians highlighting equity concerns to support treatments they think are clinically valuable.  Conversely participants recognised that the legislative and procedural constraints faced by the DoH and decision-makers including transparency, fairness and consistency mean that these equity narratives may be disregarded as they are perceived as unclear, inconsistent with HTA guidance or irrelevant.  These opposing framings of equity can make discussions on the consideration of equity tense, frustrating or combative. | “… one of the barriers…in terms of equity for some of these vulnerable groups is the process itself. So having to be company sponsored to initiate the process, particularly with children is a huge barrier. Because the companies are not interested in doing it.” Participant 10  “I think when it's a reactive system and you've got industry providing applications or submissions, there's probably less interest in exploring the equity aspects, unless it has as of complete direct benefit to the company. Um they'll only mention it if it's going to favour whatever technology they've got… as we become more reliant on the applicant in the HTA process [we] will have less… that will be a constraint in producing HTAs that have an equity component associated with it.” Participant 9  “You know, this is really an equity argument in itself, because there are a group of people with conditions that, you know, there can be available treatments, but they [treatments] may not be entering this country nor coming through the process, and they [patients] may not have access.” Participant 1  “because of course the industry is very much interested in… if they think that this is another element that they can influence, then, to help them get a positive funding decision, then they will.” Participant 9  “And obviously, especially in PBAC, you have a, you know, a tension between industry and government, for example.” Participant 3  “I think there's a real risk, whenever you have explicit rules and companies, there'll be gaming against those explicit rules, and so…I think the gaming side of things is always something you've got to think about in this market-based, you know, sponsor-driven approach. So that's probably my main concern about having more explicit quantitative processes.” Participant 21  “you know, the government has the priority populations and they're the correct priority populations, but there's a lot of them.” Participant 14  “I think differential understandings…. I think we don't necessarily have a good grasp yet of societal preferences around these things, and I don't think we have a society that's necessarily articulated…We have a lot of pockets rather than some sort of broader understanding concept.” Participant 3  “Yeah, and I think, you know, need is, it's a really important thing that gets considered. And especially unmet need. So if there's perceived unmet need, then that's really something that do gets attention of the committees.” Participant 2  “issues where you're equalizing access…where you're acting in a way where you're trying to actually reduce inequalities. So in the health technology sense, you're giving some additional value to the technology, saying, as I just did with the example of a product which doesn't require refrigeration, what we're saying in this case, that would enable access to a drug which they wouldn't otherwise have access to.” Participant 15  it's not an equity about a disadvantage, you know… it's more like, well, this drug works and there's an equity of access system situation there where, you know, people could be benefiting earlier… So there's things like that as equity issues that are not so much a poor, homeless, ethnic… but it's, it's more around, you know, ability to access at what point in the treatment cycle.” Participant 5  “right now we have to follow the process and follow the guidelines and, you know, to ensure equity of decision making, because there's that too, right? So there's equity in terms of, you know, equity in terms of priority populations, but then there's also equity of decision making and to be seen to be making fair decisions.” Participant 14  “I think it's like fundamentally important and I think it's, you know, very prominent in Australia, I think we have a very egalitarian kind of society…” Participant 13  “I guess because of the nature of the system, the HTA system that we're in, where it's quite combative, I guess equity gets weaponized in those regards, like weaponized from a sponsor perspective to try and say, you know, fund this particular thing. And then because, you know, it's an equity issue (nervous laugh) if you don't fund it.” Participant 13  “when you talk to industry people, I think they often focus on rare diseases or rare cancers. Which, you know, like I don't know that that's necessarily legitimate inequity, (laughs) or it's just that… a lot of times like what they consider rare (air quotes) is like it's rare because it's divided by a biomarker and a stage and it's like it's almost artificially rare as opposed to actually just looking at the disease incidence itself.” Participant 6  “it really does help us. It also helps us get a drug over the line sometimes when things might otherwise be a bit, you know when the financial or economics is not quite there…So sometimes there's an advantage to pushing a bit of an equity issue in that if you, you know, as a clinician, as you really feel the drug would be very useful and you just know that the economics isn't quite where you'd want it to be.” Participant 5  “I can recall the Department of Health being expressly… rude about the data, that it was completely disregarded. It actually was thought to be somewhat of a prop for pharmaceutical companies trying to receive access...” Participant 18  “if you're talking about a rare condition, you know, I think there generally is understanding across the different groups from the sponsors to the subcommittees to the PBAC, the need for these things. Sometimes if it's a little bit less clear, you know, there's probably some tension between the sponsor trying to build a case for equity, for a particular condition or medicine, and the perceptions about the need for equity in that space from the subcommittees and the PBAC and probably the Department of Health to some extent as well.” Participant 20 |
| 2.5 Disconnect between technocrats and the public | HTA decision makers are seen as technocrats appointed by the government to act on behalf of the public interest. Participants felt their decisions around equity should reflect societal values.  A variety of participants felt there is a disconnect where information on equity priorities does not travel from the public to the technocrats through either societal preferences research (seen as not yet well established), or via government or parliamentary processes.  Whilst the Government is generally supportive of equity, their directives or guidance was seen by many HTA actors as not clear or strong enough to support decision-makers to actively integrate equity concerns.  Facing this imperfect information, decision-makers cannot effectively incorporate equity considerations into their decisions and the value placed on equity and outcomes by HTA actors and technocrats may not align with the values of the public.  Instead, decision-makers must make assumptions on how to value equity. This can make decision makers feel uncomfortable as they balance a sense of responsibility to act upon equity concerns but do not feel adequately equipped (with evidence on public values) or supported (by clear government directives). Concern with this perceived overreach can diminish the impact of equity on decisions. | “I think that we're making decisions on behalf of the community, and we have to reflect… as far as possible community values. So if the community is in favour of equalizing health across the population, which I think in general it is, that should be reflected in HTA as long as the evidence is there to do so.” Participant 12  “I think we've got a disconnect between society and their voice through politicians, the technocrats, if you like, that sit within this kind of space, and then the research to actually be able to provide the support to the technocrats that actually links back to the public preferences or their voice through parliamentarian process.” Participant 3  “some of that hasn't necessarily been satisfied to a point where… yep, we've confidently got equity weights that are accepted and understood… And certainly we don't have that accepted or approved (air quotes)… if you like, or accepted by our decision makers… and be able to make decisions confidently... therefore, you know, that's a very early stage, if you like, in terms of knowledge. ” Participant 3  “I think if, so say the, the PBAC guidelines got updated tomorrow and said that, you know, we want you to systematically consider equity as part of your submissions. Um, and these are some ways you could do it. Yeah. Like I don't, I don't think there would be political objections to that…” Participant 6  “I don't think we have clear goals in our system. I don't think we have clear alignment or consensus on what we're actually trying to solve for in the first place. And then that kind of like blows through to this mess that we've got. [laughs] So for me, we'd be starting with an agreement on what…what we want the system to achieve, in terms of like access and equity. And then, you know, the system would kind of like flow on from that to ensure that those goals are met.” Participant 13  “And then government, um (hesitates) again, you know, from the HTA department, you know, the Department of Health, again, I feel there's very, (hesitates) it's really hard, it's all, you know, it's very sort of, wishy washy, there's no concrete…there's no… they'll really take our lead. So they'll very much take our lead as to where we want to go and how far we want to push it as far as people on the committee.” Participant 5  “the HTA review is quite emphatic about Aboriginal and Torres Strait Islander people. So I think we will find out, with this government, whether they do implement these policies in there, exactly where their priorities lie.” Participant 21  “I think healthcare has like a bias towards like particular outcomes. And we say, well, this is really good. And, and when you actually speak to the patient, they're like, well, I don't care about that. (laughs) So there's like a massive disconnect.” Participant 13  “my personal opinion is I think, again, that this should be debated in parliament, as they’re our representatives for the whole of Australia. And I think there's a political unwillingness to do that. And as a result, it's left to the Department of Health and a select member of individuals on a committee. Which may or may not be representative of the Australian population.” Participant 19  “It's surprising how little we do sometimes know about distributional elements of some of the conditions that we look at. A lot of assumptions are made about access, which are not necessarily correct in terms of how people access things.” Participant 15  “that's the biggest barrier really. And I mean, it stops you, you know, I'm very concerned about um… stereotyping or placing, you know, my opinion over, you know, what might be. And so I think it does sometimes, you hesitate sometimes to go too heavy on the equity aspect because of that.” Participant 5 |
| 2.6 Establishing societal values is difficult | To meaningfully integrate equity (qualitatively or quantitatively), we need to more clearly understand public values or preferences as to which social groups should be prioritised, and to what extent equity should be prioritised over efficiency.  Determining these public values regarding equity is seen as methodologically and ideologically challenging.  Aggregate or mathematical methods such as discrete choice experiments can be used to generate societal preferences for equity but participants note these are static and question whether these are robust, transparent and representative.  Deliberative approaches such as Citizen’s juries were seen as an alternative way to incorporate public values.  How equity impacts resource allocation decisions should be made understandable to the public but public comprehension of HTA broadly is poor, making public engagement challenging.  Reaching consensus on equity priorities is thought to be difficult as people have wide ranging beliefs on equity and how this might impact the use of taxpayer dollars. This plurality of values makes establishing societal values controversial and politically challenging.  Equity is a normative concept and depends on the social and political environment. There can be differing social norms around which social groups are seen as ‘worthy’ or are the responsibility of the healthcare system to address. For instance, there are concerns that stigma can deprioritise underrepresented groups.  There may be reluctance among HTA actors to obtain public preferences as once established, we are obliged to follow them even if they don’t align with our own perceived equity values. | “what are the main challenges we face to systematically and explicitly considering equity in our processes at the moment?”  “I think differential understandings. So I think we all hear these things and have a different understanding of what that is or what that might be. I think we don't necessarily have a good grasp yet of societal preferences around these things, and I don't think we have a society that's necessarily articulated…We have a lot of pockets rather than some sort of broader understanding concept.” Participant 3  “I've been involved in a study where we have developed weights for a project that we did. And it was more of an academic exercise. It wasn't something that was never ever formally adopted afterwards. And it was really tricky to do. Right? It was really, really tricky to do…” Participant 14  “taking the average of a bunch of individuals, whether it's PBAC decision makers, or the government, or patient groups, the average over all of them, they might not necessarily be representative of what the extremes are. So, like, as in, like, one particular characteristic might be really, really important for a small group. But on average, it's not important for the group. So, therefore, it's discarded… trying to pick out those nuances of what's important on average versus what's important across the whole distribution is also very important to take into account.” Participant 19  “Whereas I think using things like Citizens' Juries, where you can help people understand what you're talking about before you start, and then have that deliberation process. And you could combine that with some of the other techniques to actually get some sense of the weight, weighting between the different methods.” Participant 15  “Well, I think it has to be, yeah, it has to be something that's defensible to the public and why you're doing it. Right. I mean, it's taxpayers' dollars, right? So we are all, all of us who are involved in all this, ultimately rationing taxpayer dollars, which is the community's dollars, our population's dollars. That's what we're doing. We're not rationing our own dollars. The government's dollars is the community's dollars. So they should be reflecting, in all honesty, community preferences, but informed community preferences.” Participant 14  “we never have a conversation with the community about some of the decision making that is happening. And the community is fundamentally unaware of it until like they have an auntie or you know, child or something that's caught up in it. So there does need to be conversation about kind of, you know, what is happening and what we do value in those decision-making” Participant 13  “I think people would be surprised to perhaps find that if you're trying to be equitable, that their interests may not be preferenced and that another group may be preferenced beyond them. And I think some of that is stuff that I think, maybe I'm sounding really old here, I just think people struggle to understand that idea, and how it actually can play out, that's potentially not in an individuals um, in line with an individual's aim.” Participant 7  “Yeah, and I think you've got to have a fair degree of political will to be able to kind of, yeah, I mean, it's a, yeah, communicating to the majority of people that we're going to prioritize in a certain instance, another smaller subset of the population is a challenging thing to do and requires substantial political will.” Participant 17  “…they you know they don't lobby. They're not like some groups you know who are really active lobbyists and you know get out there and they've got they're very polished and they're very, you know whatever…  I don't know what it is. I think some groups are just more stigmatized than other groups. … And I think we can't ignore that. And I think people with severe mental illness are highly stigmatized” Participant 14  “I think generally speaking, committees would be happy to agree that the geographic and Indigenous inequalities are unfair inequalities, and are the responsibility of the healthcare system to do something about.” Participant 8  “But whatever process it has, it has to be genuine. And so if the population says something vastly different, or different to those who have well experienced in this area, are we prepared to shift values in response to the population? Because I think it's quite challenging.” Participant 7 |
| 2.7 Reliance upon qualitative evidence to support equity | Equity evidence in HTA (if provided) is overwhelmingly qualitative. While qualitative evidence is seen as appropriate, for instance for patient experience inputs, it is also seen as less impactful and less explicit or informative compared to quantitative evidence.  Qualitative equity evidence described by participants included narratives, surveys and patient preference studies. It is difficult for this evidence to be rigorous and detailed given the constraints of the HTA process faced by patient or consumer representative groups.  Even when qualitative evidence is available, it is not well integrated into the evaluation due to a lack of HTA capacity, skills or directives.  This leaves decision-makers relying upon commentary from representative groups at deliberation as a proxy for more informative patient experience or equity evidence embedded within the assessment.  Industry and patient/consumer representatives felt this qualitative equity evidence can be perceived as less robust and assigned a lower value by decision-makers than quantitative evidence. This limits the impact of equity. | “I think qualitative can be done, you know, you could see comment on things, but I think often in that qualitative sense, it often doesn't lead to action.” Participant 16  “Well, I think with qualitative, it’s not ah, you're not actually looking at numbers in terms of health impacts. And it's more of sort of speculating I think.” Participant 11  “Because in a world where you can't accurately measure distributional impacts of an intervention, there's a tendency in certain settings for them to be of diminished importance because what you can measure becomes important.” Participant 12  “…I'm such a quant[itative] person, that when you have evidence, when you have data, it can be very powerful to say, look, look at these differences and look what's happened. I just think that is so powerful if you can get the right evidence.” Participant 16  “I think quantitative can be more explicit, potentially, and more comprehensible in some ways, you know to actually see numbers and kind of go, this is going to have twice the impact on, you know, remote people, for example.” Participant 21  “There isn't *time* to do that sort of work unless we have information available through our own studies. But we do put in …qualitative information. So what people are telling us in terms of, you know, the situation in relation to their treatment. So it could be a narrative. It could be a comment.” Participant 10  “the way the evaluation teams work, incredibly talented as they are, I don't think we have many people on the evaluation teams to know how to evaluate that evidence… another thing is actually resourcing appropriately, you know, to actually be able to have those skills. So many of those HTA teams have people with qualitative research experience in it… to make sure we've got them and that the process is actually quite clear. So guideline and staff resourcing…” Participant 7  “we really underuse qualitative research in Australia, is my experience… you could at least encourage it within the system, because then actually you don't end up using participation in the appraisal side as some sort of proxy for research. Instead, what you're doing is enabling people to participate,” Participant 7  “…have to acknowledge that the data is more qualitative than quantitative… that should be acknowledged as testimony and not discounted heavily because it doesn't fit within… the type of evidence framework that the PBAC requests, you know, randomized, double blind, placebo controlled or active controlled studies is here, testimony is at the bottom… those type of things should be listened to because ultimately, I think health equity is understanding the experience of people who live with conditions or have a lack of access.” Participant 18 |
| 2.8 Concerns with adoption of quantitative equity evaluations | Participants held various reservations with the use of quantitative equity evaluations and feel these are not yet ready for adoption.  Many participants lack confidence in equity weights based on their derivation, the potential for preferences to change over time, the inability to account for intersectionality across social groups, the limited understanding of weight among decision-makers and the need to set an ICER threshold.  Participants held fears an algorithmic approach would lack the nuance and flexibility of the deliberative process.  There were concerns that quantitative equity evaluations would increase complexity. This complexity could be a disincentive to sponsors to submit or slow HTA processes, potentially exacerbating access issues for Australians. Complexity may also mean decision makers misinterpret or give less attention to equity.  Data limitations were frequently cited, particularly for children and females. Data for Aboriginal and Torres Strait Islander peoples were seen as especially challenging to obtain with additional resource requirements and demands on Aboriginal researchers limiting progress.  These data gaps were perceived to generate greater uncertainty which may in turn reduce the impact or equity-informed analyses.  Complex methods such as distributional cost-effectiveness analysis were perceived to have limited transparency and applicability due to their implicit underpinning assumptions and may have less impact than qualitative approaches.  Equity-informed economic evaluations are seen as less feasible due to cost and time constraints and limited workforce capacity. Training would be required for all HTA actors to apply these methods.  There were strong concerns for potential ‘unintended consequences,’ particularly that quantitatively prioritising some groups could inadvertently disadvantage others, potentially worsening inequalities.  Therefore some participants believed that if quantitative equity evaluations are used, some believe they should be used in every instance for fairness.  The experiences of international HTA bodies were viewed both as reasons to avoid and to support the adoption of quantitative equity consideration. | “some of that hasn't necessarily been satisfied to a point where as a HTA kind of analyst, if you like, or trying to put something together, would be like, yep, we've confidently got equity weights that are accepted and understood by decision makers…” Participant 3  …the other problem with research that I do have a bit of an issue is, is that we, we don't reproduce things, right? So you do one study, you get results and then you move on and no one, you know. And I would have more faith, I think, in weights and those sorts of things…. if we were all independently reproducing the same results and I would have great faith, more faith.” “And updating, absolutely. And this is the other problem… you know, we should always be going back and revisiting cause things change.” Participant 14  “The male-women, I think, is a classic over the last 80 years, for example. If we ask the questions in the 1930s, 50s, versus asking now, they're very different. So changing societal preferences, but also, we don't want to necessarily lock ourselves into kind of social preferences of a particular cohort and then carry that forward. And I think there's a risk of that.” Participant 3  “in terms of when we think about ah socioeconomic groups, you know, we're seeing kind of, or the weights that I've kind of seen is, you know, there's a geographical kind of weight, you know rural versus urban, for example, but also then an income kind of weights, so those you know, quartiles of quintiles of income, but then also in terms of indigenous, non-indigenous, for example, but then that ends up, you know, creating the scenario where you've got rural indigenous and low income. And so therefore, to an extent, out of the weights that have been presented, you know, is it a multiplicative, is it an additive kind of model.” Participant 3  “…so practically we don't have a threshold for that very reason, so which kind of restricts ability to do those analyses anyway. So the extended effective cost effectiveness analysis … I can kind of show the impact, you know, alongside an ICER, …but the others are a lot more inflexible and do require a threshold, and we don't technically have a threshold in Australia. So that kind of restricts the ability to do that.” Participant_19  “I don't think we want a system that is more rigid. We want to maintain some flexibility and, you know, applying a bit of common sense and humanity. I think, you know, we don't want to be completely tied to numbers. I think we need to, you know, look at it from a broad holistic perspective as well, but, and apply a bit of common sense” Participant 20  “I mean you get industry commentary saying we're spending two million dollars on an application at the moment, so they're clearly a bit worried about the cost that they have to pay, and the investment in these, so there is a risk that this could further compound reluctance, and in that way it would actually be counterproductive for equity, because we want to make it easy for a company with a low-volume drug to actually enter into the Australian market. I'd say, because the market is not necessarily as attractive as some other markets. So you know especially when the price for low-cost drugs can really be squeezed, so you know, we certainly wouldn't want it to be a disincentive to companies to apply.” Participant 21  “So in a sense, keeping it fairly simple for clinicians and for people in the HTA system who have a lot going on and a lot to look at and a lot to process. And if there's some complicated measure, it's going to be too much, too hard, won't, you know will get skipped or done in…done inappropriately or just not at all, or wrong. So keeping it fairly simple.” Participant 5  “Well, the challenge will be whether we can get reliable data, to make that informative. Um yeah, to make it informative and robust, I think that will be a challenge, to be honest.” Participant 2  “There are, is often no data if you wanted to do it by Aboriginal Torres Strait Islander, that data usually just does not exist.” Participant 19  “yeah, I should have mentioned that as a challenge [data for Aboriginal and Torres Strait Islander people]. And that's probably why a lot of this work needs to get funded first… have Aboriginal researchers on board… but they're so busy. That's another challenge. They're so busy with other work. They're so stretched, you know.” Participant 11  “the other thing that I would be concerned about and wanting to mitigate against, would be the introduction of more uncertainty, because uncertainty as a concept is a big thing in HTA, (laughs) as you’ll be aware. So, you know, uncertainty translates to risk, which translates to, you know, I don't know, pricing issues, and so on, that translates to lower prices. And from the industry's perspective, uncertainty is always, you know, a risk, I guess. So we wouldn't want it to introduce more uncertainty and therefore kind of… impact, access, risk sharing arrangements, all of that kind of stuff.” Participant 20  “I think transparency’s important… the quantitative, they use weights to adjust and the weights are informed by some other econometric models that they got from somewhere from the data… so in a way… it may appear to be transparent, but, but it's also underpinned by some data behind, right?... ultimately it might be very quantitatively difficult, which is fun for health economists, but actually doesn't make any impact on the decision ultimately in the end… and new methods… overly complicate things, but then ultimately it might not actually change things.” Participant 2  “If the intent is to introduce numerical considerations of equity… then there would be additional need for upskilling of everyone in the system. So committee members, sponsors, evaluation groups, the department staff, everybody, which is, I suppose, another reason why ah… we have to think carefully about whether we want that full quantitative approach to be implemented” Participant 12  “as soon as you start defining exactly what you consider to be equity, for example, using multi-criteria decision analysis. If you… it effectively means that any of the criteria you don't include in your analysis are effectively ignored or given zero weight. So if you don't have Aboriginal Torres Strait Islanders in there, well, then they get given zero weight. If you don't have non-English speaking background in there, they're effectively given zero weight. If you don't have children from, with severe disabilities with high impact on carers, for example, they're given zero weight. So you have to be very careful about going down the quantitative route rather than a qualitative route because it does create less flexibility in the system and can have unintended consequences.” Participant 19  “I think it would have to be done really thoughtfully. And that's just because I think we just have to be careful that anything that goes into that evaluation is really well understood in terms of, does it create an inequity then in who can provide that data? Cause there will be communities who will not be able to provide data. Um, and, And do we disadvantage anyone in that?” Participant 7  “there's also equity of decision making and to be seen to be making fair decisions. And so often where you might see carer impacts a lot, is for things with little kids and that freaks, you know, that's always, that almost tugs at the heartstrings, but there's also carer impacts for it. I'm using this as an example. In other segments of the population that may not tug at your heartstrings as much, but equally as important. So I think if you're going to make a rule that's going to be for everyone, you know, maybe, but it's not in submissions right now.” Participant 14  “these methods as yet, at least in the health sector, are relatively novel, relatively new. We don't understand particularly well what the implication… what the unintended consequences of quantifying something might be in this process. And to give you a reason why I have that concern is, the national, NICE recently introduced… a severity modifier… And it had an unintended consequence that… actually led to decisions which weren't acceptable, basically…. just saying we need to be aware that these are the consequences around these things.” Participant 15  “…NICE, I'm not sure if you're aware, but they've changed their guidelines recently, and allowing socioeconomic position, the quantification of those inputs in those groups… the fact that NICE are leading the way in these methods… and it's more of a, ‘okay, it's been done before, it's not reinventing the wheel’ kind of thing. And, you know, we do look at NICE, I guess, for that kind of role model.” Participant 11 |
| 2.9 Conditions supporting the impact of equity | Equity does meaningfully impact decisions in some instances. The listing of vaccines for Aboriginal and Torres Strait Islander people was the primary example cited.  There were specific conditions described in this situation that helped support the active integration of equity:  There is broad consensus that reducing inequalities for ATSI people should be a priority.  Decision makers are provided with quantitative equity relevant evidence including differential uptake, effectiveness and disease burden across social groups.  A more prescriptive appraisal structure is applied in the evaluation of vaccines. ATAGI uses the ‘GRADE evidence to decision framework’ for decision making which includes equity as an assessment criterion.  There are funding structures present for vaccines that enable equity-focussed decisions targeted at particular social groups (listing for Aboriginal and Torres Strait Islander people).  Decision-makers must consider budget impact. It is easier to assign value to reducing inequalities when the budget impact is modest such as for targeted funding for relatively small Aboriginal and Torres Strait Islander populations. | “There are some examples in PBAC decision-making where the decision has been, ‘this is a particular issue’. So meningococcal, MenB, meningococcal B vaccine, that was the rationale for a listing for MenB for Aboriginal, for First Nations people but not for the broader population. We accepted a higher cost effectiveness for that particular population because we felt there was that higher need.” Participant 15  “I think that the inequality that's given the greatest attention is the First Nations one, but I think that that's justified. There's a deep, long-standing history of inequity between the indigenous and non-indigenous population, and therefore it's priority number one, and it ought to be.” Participant 12  “So all the analyses are usually stratified by Indigenous status. And that would mainly be vaccine coverage.” Participant 1  “but it's not… you know very rarely measured. I think, again, vaccination is probably the standout where we have. Again, because of the real focus on the indigenous population and equity there, is where there's probably a lot more of a quantitative aspect, but the vast, vast majority is very qualitative.” Participant 5  “for example, in the GRADE evidence decision framework… So an example ATAGI would follow that process and …Equity is an item in there… it's in the evidence decision framework criteria, so it's, when ATAGI make a recommendation for a vaccine they have to consider, you know balance of safety, efficacy, acceptability, equity is one of those…we're clear as to why we've made that decision and there is a checklist of those considerations, that includes equity.” Participant 1  “I've never seen… very, very rare except again in vaccination because vaccination I think is almost a different situation because the issue particularly around the Indigenous population is built into the system there because of a different vaccination list for the Indigenous population. So they kind of have to address that.” Participant 5  “I mean, there's lots of examples of vaccines, obviously, a lot of the programs are designed with equity considerations in mind, and in particular, as an example, ah, funding for ah eligibility criteria for vaccines for Indigenous people is often a small but important subgroup.” Participant 1  “I do know that PBAC will often consider a higher shadow price for [technologies with an equity benefit], but I don't know what that price would be or how they come up with that. Often, I guess, thinking of Indigenous people as a, you know, as a specific group, they're relatively small, so the budget impact's not big. So I think PBAC often just let things through if it's a disease that's known to be disproportionately, where the burden disproportionately falls on First Nations people.” Participant 1 |

**Theme 3: Incremental and pragmatic HTA reform**

| **Subtheme** | **Brief description** | **Supporting quotes** |
| --- | --- | --- |
| 3.1 Maintain a deliberative process | Participants prefer to maintain a deliberative decision-making process moving forward. Many also preferred for equity information to remain predominantly qualitatively.  This approach is preferred as it is more feasible, avoids the need to set an ICER threshold, and is more palatable, avoiding political risk. Maintaining flexibility was also critical.  The limitations of relying upon qualitative equity evidence were acknowledged, therefore participants saw scope to:  - include equity- informed economic evaluations as an additional input (separate to the base case)  - use extended cost-effectiveness analysis (ECEA) to analyse equity impacts such as out-of-pocket costs alongside but separately to the cost effectiveness analysis. ECEA is seen as more flexible and has been more flexible than DCEA, avoiding normative judgements, and has been applied in other contexts (health policy or state government HTA).  - use subgroup analyses by equity categories was raised, but it was noted this can potentially create equity issues  -generate a more established body of research to support adoption of equity-informed economic evaluations  - use case studies or pilots to demonstrate whether the application of equity-informed economic evaluations is feasible and impactful. Starting with case studies in more socially acceptable equity categories to facilitate uptake. | “…I think that the more…qualitative contextual consideration by a group of experts is a good way of capturing nuance across submissions…” Participant 12  “I think that the way it's done now in those sort of…less formal, more narrative kind of way, is good (some hesitation) because it allows some flexibility. So I mentioned, you know, they accept higher ICERs for rare disease, for example, and that's at the discretion of the committees. And I think everybody appreciates having that flexibility built into the system. And I think everybody recognizes the need for that flexibility.” Participant 20  “I think that (pause) there is a political sensitivity associated with a more mathematical way of equity weighting. So if we actually say, we value QALYs that accrue to a particular population 50% more than we do to another group, and that gets rubber-stamped by government, that is a political risk” “handling it more qualitatively um (pause) avoids that specific risk… ” Participant 12  “you've got your base case, which should be very much guideline driven. And then if you feel there's really special, you know, there's other special considerations that need to be considered, for example… it could be a distributional cost effectiveness analysis. That's where that should be done... And then in a secondary analysis… I think then you can present alternative scenarios that highlight particular important considerations for your intervention.” Participant 14  “So the extended effective cost effectiveness analysis where I can kind of show it in like different, (hands indicating balancing) for example, I can show the impact of the ICER or costs or QALYs by different groups, or I can kind of show the impact, you know, alongside an ICER, like is a lot more flexible, but the others are a lot more inflexible and do require a threshold, and we don't technically have a threshold in Australia. So that kind of restricts the ability to do that… I just want to reiterate, I think qualitatively or extended cost effectiveness analysis is probably the better approach.” Participant 19  “We've looked at to a certain extent, certainly in terms of ideas of extended cost-effectiveness analysis, certainly in terms of thinking through issues around out of pocket cost, for example, and primarily thinking through the lens of equity of access… We were looking at, for example… adherence, medication supply, as well as services, service access, time of, or age of treatment, preceding services for hospitalization and a few other kind of metrics to try and include, that alongside, if you like, cost effectiveness rather than a distributional cost effectiveness analysis per se.” Participant 3  “We're less able to do, or less encouraged to do, those analyses by different equity levels because actually it's causing the opposite effect where it's, it's restricting access to certain patients who you would think would actually be able to benefit more but actually they can't due to lack of data… it has been having unintended flow on consequences last 20, 30 years. And that was raised during the Senate inquiry called the Zimmerman report... And so as a result, government policy has changed on these factors.” Participant 19  “so if I'm doing work in [disease]… for example the EQ-5D might be okay it's been shown to be pretty sensitive, but if I'm doing work in [subset of disease] I wouldn't use an EQ-5D, I'd use… another instrument that has been developed, you know, collaboratively with people, you know, it's been co-developed… So I feel pretty comfortable using that as an outcome measure… So I think all of that research helps to, you know, have greater confidence in the use of these things.” HTA6  “There needs to be some examples that run through the committees that show everybody the value of using more explicit equity considerations in the analysis.” Participant 8  “there could be a piloting of some tools to say, we're going to do X many cases… maybe that would be a way forward. And then I would hope that seeing it work, there would then be more appetite for it to happen again… I think sort of the beginning of more fleshed out equity consideration in these committees would start with something less controversial like the geographic inequalities, um because I don't think that's going to have… the same barrier. So that's a good start…” Participant 8 |
| 3.2 Desire for incremental change | The current HTA review process was viewed as a catalyst for potential change in the way equity is considered.  Participants described that changes could occur within the assessment process (producing better equity evidence through prescriptive HTA guidelines), in decision-making (using a framework or checklist to make decisions more explicit and transparent), or both.  It was acknowledged that maintaining deliberative processes with qualitative equity evidence (similar to the recommendations in the HTA review) represents incremental change or ‘baby steps.’  This pragmatic approach was supported because:  -it is more feasible  - HTA reform (especially significant reform) takes time  - Equity is a pressing issue, requiring urgent action so taking imperfect baby steps is preferred (to slow wide reforms)  - Substantial HTA reform, such as the quantification of equity values, involves high stakes choices with potential uncertainty, political ramifications and unintended effects on industry.  - Responding to quantitative equity evidence may be confronting or challenging for decision-makers.  - HTA actors may be risk-averse, constrained by legislation, precedence and public or stakeholder perceptions of their decisions.  There is a conflict in opinions between:  - the mandatory inclusion of equity for every HTA submission (for fair and systematic decision-making processes),  and  - wishing to avoid additional burden (of including equity information) that may deter sponsors from entering the Australian market.  To overcome this a triaging approach undertaken early in the HTA process could be used to identify submissions where a more comprehensive equity evaluation might be warranted.  Converse to the prevailing desire for incremental change, some participants argued that wider HTA reform is needed such as:  - expanding capacity to evaluate a wider range of potentially useful technologies.  - addition of a proactive approach to HTA (whilst still maintaining the reactive or sponsor-driven system) was seen as a way to reduce inequalities in a targeted manner. | “I think the methods review of HTA has sort of spurred on the motivation to actually get something concrete that could be used, going forward… just talking about ideas… we…about that calculator used for socioeconomic position.” Participant 11  “So if it's [equity] coming in at appraisal, I think we need more transparency around how it's used. And one of the ideas that sits in the HTA review is to have a qualitative values framework… so that people understand what sort of information they're looking for there, but also so that you could, in a simple way, reflect how the different pieces of information and different values were balanced at appraisal… I just don't think that there's, there's evidence coming through to, to actually support equity most of the time. So if you want to increase equity in your health system, I'm not sure the evidence that comes into HTA prior to appraisal actually helps you to do that. And HTA needs both.” Participant 7  “I think that's similar to what I was thinking before, just having something consistent. It's a good first step. It's achievable. It doesn't take a lot. And so therefore I'd say that those guidelines were pragmatic. The recommendations, sorry, were pragmatic.” Participant 3  “…it seems like it's a really simple thing to implement. And I think it would make a difference is just having a qualitative statement in there. You know, it’s just…It's not perfect. I think again, what they've suggested is a much more structured, better, long-term thing. But I mean, honestly, we could put in a qualitative statement tomorrow and it wouldn't take time or money particularly. And I think it would have an impact and it's not perfect, but I don't think we should not do it because it's not perfect. It still has some impact, because it's better we do something than nothing… cause it might take a few years to get a qualitative framework up and running and formalized and accredited and checked and, you know, and all of those things” Participant 5  “No, I think that [recommendations in the HTA review] would be a good way forward. I think you have to take baby steps. I can't think of another better proposal. Um…So, I think that would be a very good first step in the right direction. Yeah.” Participant 4  “of course the industry is very much interested in, in this element, where it favours them, so that they, if they think that this is another element that they can influence, then, to help them get a positive funding decision, then they will… It's definitely in their minds. I know government is well across it. And this is a pure example.” Participant 9  “but if we go down that path, how on earth are we going to make a decision at the end of that time? …how is MSAC going to use this information? How's it going to play into the decision making?... Because the more you acknowledge it, the more you say, yeah, that's a problem... Hmm, what are we going to do about that? And that's the problem, that people might not necessarily be willing to acknowledge, and yet live with the inequalities, being more knowledgeable about it.” Participant 8  “And I'm inherently conservative. So when I say conser[vative]… not politically conser[vative]”  “No, no, but because of risk.”  “Yeah. Risk averse. Especially when you're (pauses) in cost-effectiveness. Yeah. So conservative modelling assumptions, you know, all of that sort of thing.” Participant 14  “Then there's ah PBAC and MSAC decision-making (pauses) is influenced by precedence, what other things have happened before, and then trying to keep consistency in decision-making, because that's where you sort of, if you're inconsistent in administrative law, that's when you get into trouble. So consistency is really important in decision-making. So there would be, so if you start to do this, you have to also, start to have some sort of consideration. You can't say, well, we considered, formally considered equity as an issue here, but we're not gonna consider it here. Once you've done it, then you have to be prepared to accept that information and demonstrate that you've looked at it in a consistent way as you go forward.” Participant 15  “It'd be nice at the sponsor level that they would be required to put in a statement about equity, in the very early stages… I have no problems asking particularly the sponsors to do lots of work, you know, in the sense that they want their drug, you know, you want the best outcome. So there's something they can apply…” Participant 5  “you know, one of the recommendations in the HTA review talks about having sponsors having to put something specifically around the impact for Aboriginal and Torres Strait Islander people. I don't feel like that's an issue but I've heard some sponsors kind of go ‘uhhh that sounds a bit annoying, and just a whole extra thing we have to do.’ ” Participant 21  “I imagine in the MSAC space, you could do it at the PICO stage potentially… you'd have some sort of equity section that says, this is something that we need to do more analysis on… at least to start putting in there a bit more formally for some cases. So you wouldn't necessarily go, maybe you'd have initially a triage, but you'd have a very high bar to say, look, this could really have this potential to really exacerbate inequalities. I think we need to formalise some sort of assessment…” Participant 16  “I think we need to reform the pathways so that I think like, I think there would be just one pathway… for everything. And then like a way to kind of like deal with that in a pretty sort of proactive away… more meaning like you would have like a single front door… like a system that's built with a goal in mind, a system that's got reactive/proactive elements, a system that's like an open front door, it doesn't throw things out because it's a particular type of technology, um digital or whatever.” Participant 13  “But if you are doing in a proactive HTA system, where you are deciding what technologies you want to bring in or to assess. You might want to also consider that at the prioritisation stage, where you're working out what sort of um, technologies you want to… actively investigate and invest in for.. to.. you know, even things up.” Participant 9 |
| 3.3 Look beyond listing | Participants believed we must also look beyond the decision to recommend or list a technology in order to reduce health inequalities.  An equity lens should be applied across all stages of HTA including:  1.  Agenda setting- Adopt a proactive approach – introduce an agenda setting stage to target technologies that reduce inequalities.  2.  Establishing equity relevant safety and efficacy data (pre-HTA) can improve the consideration of equity  3.  The administration of the PBS or MBS after listing can create equity issues. For example:  - burdensome restrictions are not reviewed with equity or fairness in mind.  - retention of technologies (delisting decisions) and disinvestment decisions lack an equity lens.  4.  The equity impacts of HTA decisions could be evaluated post-listing, gathering evidence on uptake and effectiveness of funded interventions across social groups. This information could inform future decision-making.  Participants briefly discussed HTA outside of PBAC and MSAC pathways such as health program funding decisions. These processes were seen as:  - less structured - equity can be more freely integrated, and is sometimes requested.  - ad hoc, less transparent and open to influence  -supported by lesser HTA capability  Equity consideration in resource allocation decisions at the state level are also important to understand.  There is support for extending the PBAC/MSAC model of HTA to other health program or prevention funding decisions.  An intersectoral approach to policy-making is needed to address health inequities. | “I've talked pretty much exclusively about PBAC, but I think one of the, the conversation today is about technology assessment more generally. And I think that that's, that's one, to me, one of the great unknowns, which is how things outside of the PBS and MBS get approved and the degree to which distributional impacts have…have an impact on how those decisions are made.” Participant 12  “I think the point that it's often not the technology, the availability of the technology itself, but the, actually getting to the diagnosis and then getting to the right specialist to prescribe that are often the issues. So yeah, it might not…having the treatment available might not necessarily address the inequity.” Participant 6  “With the HTA review, one of the goals is to try and make our system a lot more proactive than what it is currently. The ah…states are good at being proactive, but they don't have a great deal amount of HTA capability.” Participant 9  “The second one is where an equity issue arises…even before the technology assessment through the registration process. So the most common one in that regard is where a drug is registered for a particular population... But by the registration process and therefore the HTA process that flows from that, you can induce an equity, an equity issue…one of the problems about the registration issue is, it's not up to the HTA, it's not up to the MSAC or PBAC to change the registration conditions, and it becomes more problematic.” Participant 15  “it's a very slow process… advocating for some system change… with biologics for example…. It's a process for people to access them because they have to go through this stepwise process of trial and error, and failing certain treatments to get them. Which is an equity issue in itself. And then the clinicians are having to go through a process of every six months, having to fill in all this paperwork… we've been advocating for these… to be streamlined and … you know, that's happened [for this drug] in some conditions, but not in others. So, you know, where's the equity in that? So there's a lack of equity in their process themselves.” Participant 10  “so there's been some real challenges in supporting drugs with low volumes, old drugs, for example, in that pricing aspect to support these products for being viable and we've seen a lot of products in the last few years over the counter products, which are mainly for Aboriginal and Torres Strait Islander people, fall off because the…within the pricing structures that are there, either in a negotiation process when they're trying to list a product, or when it's already listed but the price is being eroded, that there's not a due consideration of equity in my opinion for those products, and that's why they've essentially ultimately been delisted.” Participant 21  “We have the MBS review, which is a rolling review where they disinvest, but I don't think that they look at equity, particularly, least not as far as I'm concerned. It seems to be more about the vested interests of clinicians, actually.” Participant 9  “from an equity point of view, it looks like we're doing right things, listing things. I think a lot of the inequity comes after it's listed, right? So it's the unintended consequences… I think it's a really important area… And I guess that would then inform future decision making saying, look, what we found with these decisions…actually, and so then it would feed back into then I guess earlier decision making.” Participant 16  “so within probably the public health kind of sphere of policies, and certainly within drugs and alcohol, I'd say there's a much stronger leaning towards understanding, accepting the value, or at least being able to understand, the impacts for low socioeconomic and Indigenous populations within the broader policy space… So that would almost be a specific requirement, compared to, us just proposing it and hoping.” Participant 3  “Community-based preventative interventions, things in schools, right, that are cross-sectoral and there's no easy funding mechanisms for those now, and it's lobbyists and it's you know people that go to treasury you know um, that are well organized and they'll they might put in a business case or something and then it might may or may not get funded, or the minister has had personal experience of something and so will have their pet interests, you know, so anything that's kind of funded outside of those two formal mechanisms I think is, that’s problematic.” Participant 14  “I mean, yeah, certainly for other like aspects of government programs, services, I mean, there's huge transparency issues. I mean, I mean, you're lucky if you ever see an evaluation of a government program, it will always be done kind of, you know, if it's done, it's done kind of… well after the fact. And you know, it might be very highly sanitized…. It's not anywhere near as like, I think the whole HTA PBAC/MSAC process is like, that's like really quite a rigorous process that is very, as far as I can tell, is like quite, um, you know, relatively transparent… but when it comes to just general governance of which programs they sort of decide to fund and what they don't, like, there's not a lot of transparency around it and how they make decisions, (shrugs shoulders) no idea.” Participant 17  “… how new services are delivered, things that are done typically at the state and territory level, I don't have good visibility on that. And I imagine that the system is a bit more ad hoc and a bit patchier. So that, to me, is a big unknown…consideration of how it's done at the state and local level is extremely important…how we make decisions there probably have a bigger capacity to close the gap between...between more and less advantaged groups. So, you know, getting a sense of that's really important. Participant 12  “Which the Productivity Commission picked up in that report, especially for prevention, right?... Very supportive, right? To develop a framework, like a HTA kind of framework. I've never understood why they don't. NICE does it, right, in the UK. NICE is much broader. So why can't we have an Australian NICE…?” Participant 14  “Well, it kind of needs to be all sectors really… there are certain inequities that manifest in the health space that are… at their core driven by factors which are beyond health. So this is the more broader sort of… socioeconomic determinants of health. So I think equity should be a whole of government priority. And I think when other departments look to evaluate measures or policies that they might want to introduce, there needs to be a kind of a kind of a consideration on equity. So yes, it can't just be a health thing.” Participant 17 |
