# Supplementary material for: How Is Equity Considered Within National Healthcare Priority Setting? A Qualitative Study of Australian Health Technology Assessment Actors' Perspectives
Source: Health Expect. 2026 Oct 30;29(6):e70955. doi: 10.1111/hex.70955 (PMC13722783; doi:10.1111/hex.70955)
Supplement: Supplementary file 3 [file HEX-29-e70955-s003.docx]

**Supplementary File S1: The Australian health technology assessment (HTA) process**

Two independent, expert HTA bodies are appointed responsibility for evaluating new technologies for Federal government subsidy. The Pharmaceutical Benefits Advisory Committee (PBAC) assesses medicines for listing on the Pharmaceutical Benefits Scheme and vaccines and the Medicare Services Advisory Committee (MSAC) evaluates medical technologies and services provided through the Medicare Benefits Schedule (MBS).

After (or parallel to) registration by the Therapeutic Goods Administration (TGA), industry or clinician sponsors can submit to the Department of Health (DoH) for funding, where submissions are reviewed through the PBAC and/or MSAC pathways, depending on the technology.

This is an interactive process between government-contracted evaluators, sponsors, and HTA subcommittees: Economic Subcommittee (ESC), Drug Utilisation Subcommittee (DUSC), and PICO advisory subcommittee (PASC). The Australian Technical Advisory Group on Immunisation (ATAGI) advises PBAC on vaccines, while ESC and DUSC provide clinical, health economic, and budget impact advice, respectively, to PBAC and MSAC committee members.[1, 9, 10] During this review, the public can comment via an online portal.[11]

PBAC and MSAC decisions to recommend, defer, or reject a listing are provided to the Minister for Health, Disability and Ageing, followed by financial agreement negotiations between the sponsor and government. Post-listing, DUSC may evaluate the real-world usage and budget impact of technologies. [9] Public summary documents (PSD’s) are regularly released to transparently communicate the recommendations made.[12]
