# Supplementary material for: How Is Equity Considered Within National Healthcare Priority Setting? A Qualitative Study of Australian Health Technology Assessment Actors' Perspectives
Source: Health Expect. 2026 Oct 30;29(6):e70955. doi: 10.1111/hex.70955 (PMC13722783; doi:10.1111/hex.70955)
Supplement: Supplementary file 4 [file HEX-29-e70955-s004.docx]

**Supplementary file S3: INTERVIEW GUIDE**

For the purposes of this conversation, I would like to provide some definitions and introductory information.

We define HTA as any evaluation of the best available evidence about the quality, safety, efficacy, cost-effectiveness and total costs of a health technology (health-related goods or services) with a view to making decisions about the funding of new health technologies. A health technology, or intervention, can be a test, device, medicine, vaccine, procedure, program or system to prevent, diagnose or treat a condition, or to promote or improve health.

We define health equity as the absence of differences in health outcomes seen across social groups that are avoidable and unfair. Inequities are determined by the conditions in which people are born, grow, live, work, play and age, as well as biological determinants.

I will provide some brief information on the methods for considering equity within HTA to facilitate our discussion.

Broadly there are two approaches to considering equity in HTA:

Qualitatively: through deliberation and discussion of equity relevant information at the point of decision-making.

- This may be unstructured, or structured following some sort of framework or checklist of criteria to be considered (for example multi-criteria decision analysis (MCDA))

Quantitatively: through numerical evaluations that measure the size of the equity impact of an intervention on specific subgroups. These are sometimes called equity-informative methods. Some examples from the literature include:

- weighting outcomes or incremental cost-effectiveness ratios to account for equity concerns (equity weighting), such as a multi-criteria decision analysis (MCDA) where the criteria on the checklist are weighted by preferences or importance (called quantitative MCDA)
- evaluating non-health outcomes that impact equity such as out-of-pocket costs (called extended-cost-effectiveness analysis)
- evaluating the distribution or spread of costs and outcomes across equity groups (called distributional cost effectiveness analysis).

We will now commence recording.

| **Broad questions (must be asked)** | **Optional prompts or follow-up questions** |
| --- | --- |
| **1. Role and Demographics** | |
| Which organization do you work for, or represent? |  |
| Tell me about your current role, and how your day-to-day activities relate to HTA? |  |
| How many years have you been working in relation to health technology assessment? | (Note this information will be grouped into age brackets: 0-5 years, 5-10 years, 10-15 years, more than 15 years) |
| **2. Experiences of incorporating equity into HTA** | |
| Have you had any experience with considering health equity within HTA, and if so, in what format? | - How was equity considered? Quantitatively or qualitatively? - At which stages of the HTA process was equity considered? - Who requested the equity evaluation? - What difficulties did you encounter? - Have you ever felt you needed to change your analysis/decision/submission (choose relevant for participant) to keep things simple or save time? - How were decisions made about how to undertake or assess the equity information? |
| What methods for evaluating equity considerations are you familiar with? | (Mention the list of potential methods if needed to extend the dialogue) |
| **3. Definitions, motivations and opinions regarding equity** | |
| What do you think about the inclusion of equity considerations in HTA? | - Do you think equity should be considered within HTA processes? Please explain why/why not - Does this differ to the views of your organization? |
| For whom do you have equity concerns? Or, which underserved populations are of concern? | - For example: severe or rare diseases, gender, children or older people, race or ethnicity (ie First Nations people), socioeconomic status, LGBTQI+ (lesbian, gay, bisexual, transgender, queer/questioning, intersex and/or other sexuality and gender diverse people), people living in rural or remote areas, people with a disability or mental illness - Among these, are there particular groups for which you prioritise or hold greater concern? |
| From your perspective, is there a particular type of inequality that is receiving more attention than others? | - Why do you think this is? |
| How do you think equity impacts should be measured, what outcomes should be used? | - Some examples of outcomes from the literature include: access to healthcare, measures of health status such as life expectancy, health-related quality of life, or health adjusted life expectancy (HALE) or quality adjusted life expectancy (QALE) |
| **4. How the integration of equity into HTA can be operationalised** | |
| Do you think equity should be further incorporated into the HTA process? | - If so, how? At which stages? - (Discuss how this might look at each stage):   - identify the PICO,   - clinical evaluation,   - economic evaluation,   - considering additional elements   - Pre/post-HTA e.g. drug utilisation sub-committee activity |
| Would you like to see equity considered qualitatively or quantitatively? | - Why? |
| From your perspective, what are the main challenges that exist to more systematically and explicitly considering equity in our HTA processes? | - What would be the potential training or capability needs at your organization? - How do you feel about the complexity of the equity informed methods? - How feasible is it to adopt the use of equity informative methods? - Are your peers aware of equity informative methods of economic evaluation? - What further information do we need to help us incorporate a systematic approach to equity? (whose preferences should determine decision making) - Do you feel we have capacity to include the evaluation of equity impacts within HTA processes? |
| The recent HTA review commissioned by the Australian Government has produced recommendations for an explicit qualitative values framework, which includes a checklist to assist HTA decision-makers to integrate equity considerations into their deliberations.  What do you think about this proposal? | - Are there alternative ways would you like to see equity considered? |
| If we were to adopt this qualitative framework and checklist approach, how specifically do you see this being implemented? | - Who should be involved in the development? - How would the framework integrate with the HTA process? At which stages? - Or would it be a separate consideration alongside the clinical effectiveness, cost-effectiveness and budget impact? - Would a multi-criteria decision analysis (MCDA) approach that provides a set of criteria for deliberation be useful? How should this be operationalised? |
| **5. Conclusion** |  |
| Is there anything else you would like to add? |  |
| Are there other staff in your organization, or external key stakeholders, who might like to comment on the inclusion of equity in health care decision making at the national level? | - If so, I will note them down once we finish recording. |

Thank- you very much for your valuable time and insights.

END RECORDING
